# The design space of E(3)-equivariant atom-centred interatomic potentials

**DOI:** 10.1038/s42256-024-00956-x

**Published:** 2025-01-15

**Authors:** Ilyes Batatia, Simon Batzner, Dávid Péter Kovács, Albert Musaelian, Gregor N. C. Simm, Ralf Drautz, Christoph Ortner, Boris Kozinsky, Gábor Csányi

**Affiliations:** 1 Engineering Laboratory, University of Cambridge, Cambridge, UK; 2 Department of Chemistry, ENS Paris-Saclay, Université Paris-Saclay, Gif-sur-Yvette, France; 3 John A. Paulson School of Engineering and Applied Sciences, Harvard University, Cambridge, MA USA; 4 ICAMS, Ruhr-Universität Bochum, Bochum, Germany; 5 Department of Mathematics, University of British Columbia, Vancouver, British Columbia Canada; 6 Robert Bosch LLC Research and Technology Center, Watertown, MA USA; 7 Present Address: Microsoft Research AI for Science, Cambridge, UK

**Keywords:** Atomistic models, Computational chemistry, Molecular dynamics, Computational methods

## Abstract

Molecular dynamics simulation is an important tool in computational materials science and chemistry, and in the past decade it has been revolutionized by machine learning. This rapid progress in machine learning interatomic potentials has produced a number of new architectures in just the past few years. Particularly notable among these are the atomic cluster expansion, which unified many of the earlier ideas around atom-density-based descriptors, and Neural Equivariant Interatomic Potentials (NequIP), a message-passing neural network with equivariant features that exhibited state-of-the-art accuracy at the time. Here we construct a mathematical framework that unifies these models: atomic cluster expansion is extended and recast as one layer of a multi-layer architecture, while the linearized version of NequIP is understood as a particular sparsification of a much larger polynomial model. Our framework also provides a practical tool for systematically probing different choices in this unified design space. An ablation study of NequIP, via a set of experiments looking at in- and out-of-domain accuracy and smooth extrapolation very far from the training data, sheds some light on which design choices are critical to achieving high accuracy. A much-simplified version of NequIP, which we call BOTnet (for body-ordered tensor network), has an interpretable architecture and maintains its accuracy on benchmark datasets.

## Abstract (web summary)

Batatia and colleagues introduce a computational framework that combines message-passing networks with the atomic cluster expansion architecture and incorporates a many-body description of the geometry of molecular structures. The resulting models are interpretable and accurate.

## Main

There has been a revolution in atomistic modelling over the past decade, leading to the widespread adoption of machine learning interatomic potentials, particularly in materials science. A broad range of different model architectures have been proposed in the literature. These models are typically constructed to start with a descriptor (an array of numbers) to represent the environment of an atom. The key to the success of these models was making this descriptor invariant under the symmetry group of Euclidean symmetries (translation, rotation and reflection) of three-dimensional space (E(3)), as well as under the permutations of atoms of the same element in the environment^1^. Two examples of such descriptors are the atom-centred symmetry functions (ACSF)^2^ and the smooth overlap of atomic positions (SOAP)^3^. Many interatomic potentials have been built using these descriptors and subsequently used to model materials (see corresponding recent reviews^4–6^). More recently, it has been recognized that both of these methods can be understood as special cases of the Atomic Cluster Expansion (ACE)^7,8^. The key idea of ACE was to introduce a complete set of basis functions (using spherical harmonics and an orthogonal radial basis) for the atomic environment that is built using the body-order expansion hierarchy. Many previously proposed descriptors fit into the ACE framework, with the key differences being the maximum order of the body-order expansion (three-body for ACSF and SOAP, four-body for the bispectrum^9^ and so on) and specific choices of the radial basis functions^1,7^. An alternative way of defining features analogous to ACE is used by moment tensor potentials^10^, which construct a spanning set for the atomic environment using Cartesian tensors that can be expressed as a linear transformation of the ACE basis. ACE naturally extends to equivariant features and to include variables beyond geometry, such as charges or magnetic moments^11^. For a given descriptor, the atomic energy is fitted using a simple linear map^12^, a Gaussian process^9^ or a feed-forward neural network^2^. Other descriptor-based models have been built for entire molecules or structures directly, rather than decomposed into atomic contributions^13–15^.

In parallel to the development of models using descriptors of atom-centred environments, other groups explored the use of message-passing neural networks (MPNNs) to fit interatomic potentials. These models represent the atomic structure as a graph in which an edge connects two nodes (atoms) if their distance is smaller than a fixed cutoff. The models then apply a series of convolution or message-passing operations on this graph to learn a representation of the environment of each atom. This learned representation is mapped to the site energy via a readout function (see the Methods for a more detailed description of message-passing potentials). Early models in this class, such as SchNet^16^, Message Passing Neural Networks (MPNN)^17^, PhysNet^18^ and DimeNet^19^, used internal features that are invariant under rotations of the input structure.

A key innovation of the Cormorant network^20^, tensor-field networks^21^ and steerable 3D convolutional neural networks (CNNs)^22^ was to create equivariant internal features that transform (under the symmetry operations of the input) like the irreducible representations of the symmetry group and construct invariants only at the very last step. For example, features inside the network can rotate with the structure just like a Euclidean vector would. To create these equivariant features inside the network, these networks introduced a type of nonlinear operation—an equivariant tensor product that couples features via the Clebsch–Gordan coefficients—resulting in output features of a desired symmetry. The idea of coupling equivariant operations with message passing on the graph of atoms was introduced with Neural Equivariant Interatomic Potentials (NequIP)^23^ and improved on the state-of-the-art accuracy at the time by a factor of about two across multiple datasets. Several equivariant message-passing models were subsequently published (for example, EGNN^24^, PaiNN^25^, NewtonNet^26^, GemNet^27^, TorchMD-Net^28^ and SEGNN^29^). An alternative equivariant deep learning interatomic potential was also introduced recently that does not make use of atom-centred message passing^30^ and explicitly demonstrated the scalability of equivariant models to millions of atoms.

In this Article we describe a framework called Multi-ACE with the aim of unifying the mathematical construction of MPNNs and ACE. The construction can be understood as a MPNN using ACE as the convolution in each layer of the network. We set out a comprehensive design space for creating machine learning interatomic potentials that incorporates most previously published models. Previous work has identified a connection between body order and MPNNs^31^. Simultaneously with release of the preprint version of our work^32^, ref. ^33^ investigated the formal connection between message-passing networks and atomic-density-based descriptors. The authors made the connection by interpreting message-passing networks as multicentred atomic-density representations. Our work extends these ideas by defining a comprehensive design space for atom-centred interatomic potentials and by giving a detailed analysis of each of the components of the framework. We also demonstrate how previously published models correspond to different points in the design space and comprehensively analyse all components of the models.

Two recent papers have demonstrated the usefulness of the Multi-ACE design space. ML-ACE^34^ used a fully coupled, invariant stack of Multi-ACE layers corresponding to a point in the design space. They made a connection between MPNNs and ACE via the power-series expansion of the electronic structure Hamiltonian. The second recent paper, building on the preprint^32^ of this work, introduced MACE^35^, which uses a tensor-decomposed, equivariant stack of Multi-ACE layers, and showed that just two such layers could reach state-of-the-art accuracy at a reduced computational cost.

Using the Multi-ACE framework, it is possible to systematically probe different modelling choices. We demonstrate this through examples using a code called BOTNet^36^ (Body Ordered Tensor Network; described in detail in the Supplementary Information) and present a detailed study on which innovations and ‘tricks’ of the NequIP model are essential to achieving its high accuracy.

## Multi-ACE

In this section, we show how multiple equivariant ACE layers can be combined to build a message-passing model^34,35^ (see the Methods for a general introduction to message-passing interatomic potentials and the standard ACE and its equivariant version). The resulting framework encompasses most equivariant MPNN-based interatomic potentials. If a single message-passing layer is used, the framework can be reduced to linear ACE or the other atom-centred descriptor-based models.

Using ${\sigma }_{i}^{(t)}$ for a node state and ${{{m}}}_{i}^{(t)}$ for an aggregated message at iteration *t* of central atom *i*, we can define the Multi-ACE model as follows. First we identify the message with the output of an equivariant ACE layer and specify how it is used to form the next node state ${\sigma }_{i}^{(t+1)}$. The states of the atoms are updated by assigning the output of the previous layer to the feature ${{{h}}}_{i}^{(t+1)}$:

1 $$\begin{array}{ll}{\sigma }_{i}^{(t+1)}&=\left({{\bf{r}}}_{i},{{{\theta}}}_{i},{{{h}}}_{i}^{(t+1)}\right),\\ {{{h}}}_{i}^{(t+1)}&={U}_{t}\left({\sigma }_{i}^{(t)},{{{m}}}_{i}^{(t)}\right),\end{array}$$

where **r**_*i*_ is the Cartesian position vector of *i*, θ are immutable node attributes (e.g. one-hot encoding of atomic numbers), ${{{m}}}_{i}^{(t)}$ is a set of messages as defined in equation (21) and *U*_*t*_ is the update function for each layer. In most MPNNs, the *k* channel of the message corresponds to the dimension of the learned embedding of the chemical elements^16,23^. We need to extend the one-particle basis, φ, of equivariant ACE (from equation (13); Methods) to incorporate the dependence on the output of the previous layer, which can be achieved by making it an argument of the *T*_*kc*_ functions:

2 $${\phi }_{kvL}^{(t)}\left({\sigma }_{i}^{(t)},{\sigma }_{j}^{(t)}\right)={R}_{kc{l}_{1}L}^{(t)}({r}_{ji}){Y}_{{l}_{1}}^{\,{m}_{1}}({\hat{{\bf{r}}}}_{ji}){T}_{kcL}^{\,(t)}\left({{{h}}}_{j}^{(t)},{{{\theta }}}_{i},{{{\theta }}}_{j}\right),$$

where *R* are radial functions, *Y* are spherical harmonics, *T* are generic node embedding functions and *v* is the local correlation order of each layer (*v* ≡ *l*_1_*m*_1_*c*), *k*, *c*, *l* are indices, *t* is the number of message-passing layers, *j* are neighbouring atoms, *θ*_*i*_ are chemical attributes and *l* is the internal order of the spherical harmonic expansion within the layer in the one-particle basis. We have also added the index *L* to the one-particle basis to enable a different set of one-particle basis functions to be included for messages *m*_*i*,*kLM*_ with different symmetry corresponding to *L*. The one-particle basis can be further extended to encompass attention-based models by adding additional arguments to the *T*_*kc*_ function as ${T}_{kcL}^{\,(t)}({{{h}}}_{j}^{(t)},{\{{{{h}}}_{k,l = 0}^{(t)}\}}_{k\in {\mathcal{N}}(i)},{{{\theta }}}_{i},{{{\theta }}}_{j})$, where ${\mathcal{N}}(i)$ represents the neighbours of atom *i*.

We now relate the equations of the MPNN framework to those of the Multi-ACE framework. First we identify the message function *M*_*t*_ with the one-particle basis of equation (2):

3 $${M}_{t}\left({\sigma }_{i}^{(t)},{\sigma }_{j}^{(t)}\right):= {M}_{kvL}^{\,(t)}\left({\sigma }_{i}^{(t)},{\sigma }_{j}^{(t)}\right)={\phi }_{kvL}^{(t)}\left({\sigma }_{j}^{(t)},{\sigma }_{i}^{(t)}\right).$$

**Fig. 1:**
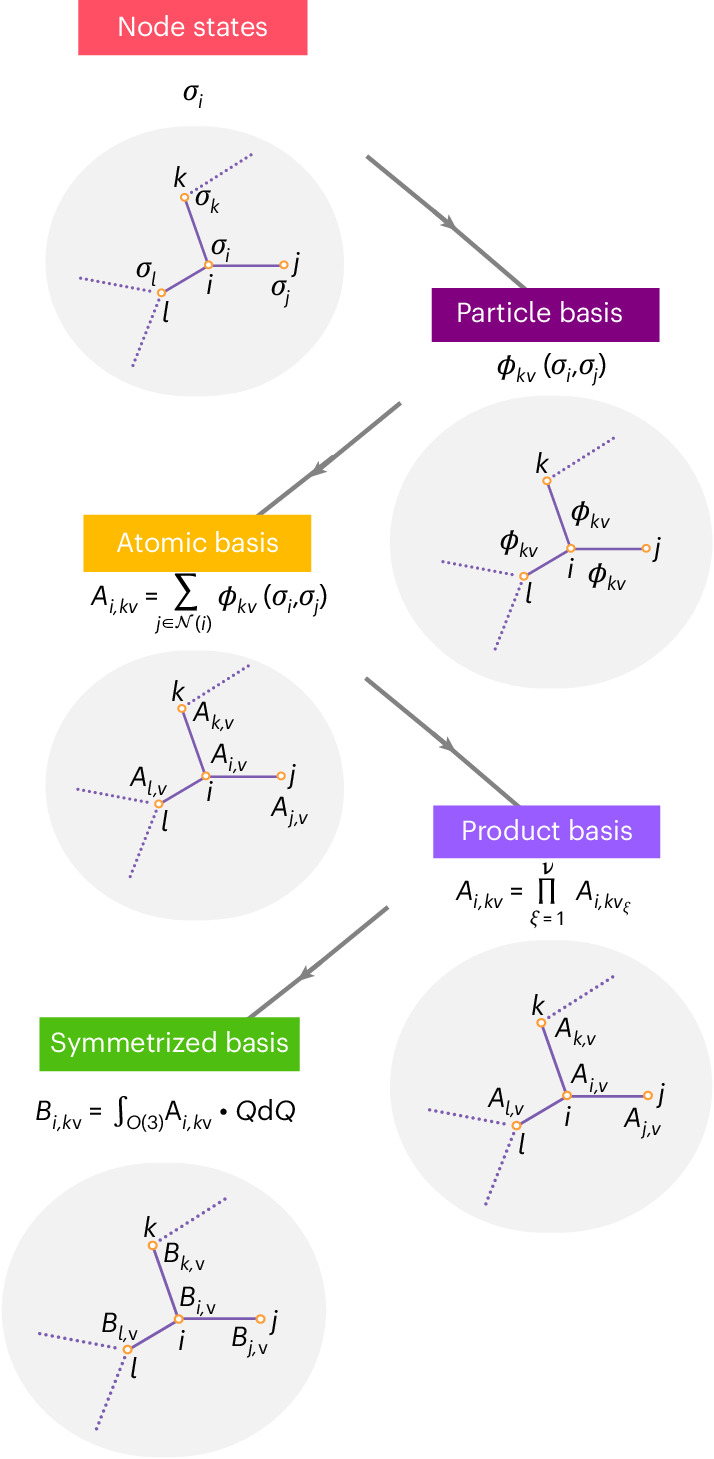
Construction of high-body-order ACE features. First a neighbourhood graph is constructed with each node labelled with its state. The one-particle basis is then computed for each edge. After that, a pooling operation is performed to create permutation-invariant *A* functions of semi-local environments. To construct higher-body-order features, the product basis is formed by taking the tensor products of all coupled indices of the *A* functions. Finally, to create equivariant messages, the *B* basis is formed by first specifying the required equivariance and then evaluating the corresponding symmetrization integral. The invariant *B* basis is shown here.

Next we define the permutation-invariant pooling operation ${\bigoplus }_{j\in {\mathcal{N}}(i)}$ of equation (23). To obtain a symmetric many-body message ${m}_{i,kLM}^{(t)}$ of correlation order *ν*, the pooling operation must map the one-particle basis that is two-body to a set of many-body symmetric features that can be combined in a learnable way to form the message on each node. This is what the equivariant ACE formalism of ref. ^7^ achieves. In this way, we obtain the central equation of Multi-ACE:

4 $$\left\{\begin{array}{rcl}{m}_{i,kLM}^{(t)}&=&\mathop{\bigoplus}\limits_{j\in {\mathcal{N}}(i)}{M}_{t}\left({\sigma }_{i}^{(t)},{\sigma }_{j}^{(t)}\right)\\ &=&\mathop{\sum}\limits_{\eta }{w}_{i,k\eta L}^{(t)}\,\mathop{\sum}\limits _{{\boldsymbol{v}}}{{\mathcal{C}}}_{\eta ,{\boldsymbol{v}}}^{LM}\mathop{\prod }\limits_{\xi =1}^{\nu }\mathop{\sum}\limits_{j\in {\mathcal{N}}(i)}{\phi }_{k{v}_{\xi }L}^{(t)}\left({\sigma }_{i}^{(t)},{\sigma }_{j}^{(t)}\right),\end{array}\right.$$

where ${w}_{i,k\eta L}^{(t)}$ are learnable weights and *ν* equals the body order minus 1. ${{\mathcal{C}}}_{\eta ,{{v}}}^{LM}$ denotes the generalized Clebsch–Gordan coefficients defined in equation (20) and η enumerates all the combinations for a given symmetry. The general scheme of higher-order message passing is illustrated in Fig. 1.

**Fig. 2:**
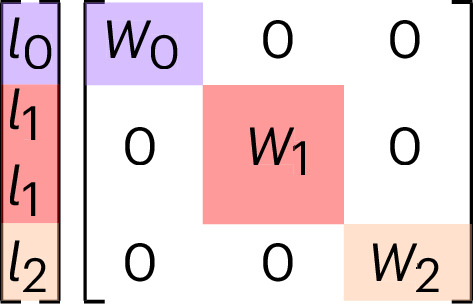
Block structure of weight matrices for an equivariant linear operation. As only linear combinations of features of the same representations (*l*_0_, *l*_1_, *l*_2_) are allowed to interact, the weight matrix is block diagonal.

The update function *U*_*t*_ from equation (24) corresponds to a learnable linear combination of the uncoupled channels of the symmetrized message and can be written as:

5 $${h}_{i,kLM}^{(t+1)}={U}_{t}\left({\sigma }_{i}^{(t)},{{{m}}}_{i}^{(t)}\right)=\sum _{\tilde{k}}{W}_{k\tilde{k}L}^{\,(t)}{m}_{i,\tilde{k}LM}^{(t)}$$

where *W* ^(*t*)^ is a block diagonal weight array (Fig. 2) of dimension [*N*_channels_ × *N*_channels_ × *L*_max_], *N*_channels_ is the number of uncoupled *k* channels in the message and *L*_max_ is the maximum order of symmetry in the message that is passed from one layer to the next. *U*_*t*_ can also depend on the attributes (such as the chemical element) of the central atom via a so-called self-connection (see below for details). The update functions acting on equivariant features can also be nonlinear, but for that to occur, the functions must have a particular form (see ref. ^22^ and the Supplementary Information). After the *T*th layer, a learnable (linear or nonlinear) readout function (that can depend on the final message or all previous ones) gives the site energy of atom *i*.

We illustrate the choices for the message function, symmetric pooling and update function for three different models in Extended Data Table 1.

### Coupling of channels

One important design choice in ACE models is how channels interact when forming the product basis. This choice affects the scaling of the number of features substantially and is therefore an essential part of the design space. This is best illustrated by considering the degree of freedom regarding the handling of different chemical elements. In the case of general linear ACE, and other similar descriptors such as SOAP, the element channel of the one-particle basis is a discrete index. When forming the higher-order many-body basis functions that will produce the features, these channels are coupled, forming all possible combinations. For example, if there are four different chemical elements, the number of three-body basis functions will be proportional to 4^3^. The alternative approach, employed by most MPNNs, is to map the chemical elements to a set of fixed-length vectors via a learnable transformation. When the higher-order features are formed during the message-passing phase, these channels are not coupled; hence the number of features does not depend on the number of chemical elements. Instead, the channels are mixed during the update phase. These uncoupled channels can also be understood in terms of a tensor decomposition of the fully coupled form as demonstrated in ref. ^37^.

Similar choices can be made for the radial basis functions. Linear ACE uses orthonormal radial basis functions and forms all possible combinations (up to truncation by the maximum polynomial degree) for the higher-order features. For example, for the three-body functions, the radial part has the form *R*_1_(*r*_*ij*_)*R*_2_(*r*_*ik*_) for all allowed combinations of *R*. By contrast, NequIP learns a separate (nonlinear) combination of radial features for each one-particle basis, as shown in equation (6). There is therefore a single learnable radial basis function ${R}_{k{l}_{1}{l}_{2}L}^{(t)}$ for each channel *k*, spherical harmonic *l*_1_, neighbour feature symmetry *l*_2_ and output symmetry *L*. The uncoupled channels *k* are mixed only during the update phase.

The analysis within the design space leads to the question of the optimal amount of coupling within the product basis in the spectrum between the full coupling of linear ACE and lack of coupling in NequIP.

### Interpreting models as Multi-ACE

The Multi-ACE framework includes many of the previously published equivariant message-passing networks. The most basic specification of a Multi-ACE model considers *T*, *ν*, *l*_max_ and *L*_max_. Other choices include the types of feature (Cartesian or spherical basis) and the type of dependence of the radial basis on *kcl* in equation (2). Note that the pointwise nonlinearities present in some of those models affect both the local correlation and the total correlation, as discussed below. For simplicity, we chose not to consider them in the following discussion. A comparison of the design choices in different models is summarized in Table 1.

**Table 1:** Different choices in the Multi-ACE formalism lead to different models in the literature

	*l*_max_	Update *L*_max_	*ν*	*T*	Total correlation order	$\boldsymbol{T}_{\boldsymbol{kc}}^{({\boldsymbol{t}})}\left({\boldsymbol{{h}}}_{\boldsymbol{j}}^{({\boldsymbol{t}})},{\boldsymbol{\theta} }_{\boldsymbol{i}},{\boldsymbol{\theta }}_{\boldsymbol{j}}\right)$	Coupling (*v*)
SOAP^3^	≥3	0	2	1	≥3	${\delta }_{{z}_{i}{\theta }_{i}}{\delta }_{{z}_{j}{\theta }_{j}}$	*nlm*
Linear ACE^12^	≥1	0	≥2	1	≥3	${\delta }_{{z}_{i}{\theta }_{i}}{\delta }_{{z}_{j}{\theta }_{j}}$	*nlm*
TrACE^37^	≥2	0	≥1	1	≥2	${\sum }_{z}{W}_{kz}{\delta }_{z{z}_{i}}$	*lm*
SchNet^16^	0	0	1	≥ 2	*T*	${h}_{j,kl = 0}^{(t)}$ (scalars)	${{\emptyset}}$
DimeNet^19^	0	0	2	≥ 2	2*T*	${h}_{j,l = 0}^{(t)}$ (scalars)	${{\emptyset}}$
Cormorant^20^	≥1	≥1	1	≥ 2	*T*	${h}_{j,klm}^{(t)}$ (spherical vectors)	*lm*
NequIP^23^	≥1	≥1	1	≥ 2	*T*	${h}_{j,klm}^{(t)}$ (spherical vectors)	*l*_1_*m*_1_*l*_2_*m*_2_
GemNet^27^	≥1	≥1	3	≥ 2	*T*	${h}_{j,klm}^{(t)}$ (spherical vectors)	*l*_1_*m*_1_*l*_2_*m*_2_
MACE^35^	≥1	≥0	≥1	≥ 1	~*ν*^*T*^ + *T*	${h}_{j,klm}^{(t)}$ (spherical vectors)	*l*_1_*m*_1_*l*_2_*m*_2_
NewtonNet^26^	1	1	1	≥ 2	*T*	Cartesian vectors	–
EGNN^24^	1	1	1	≥ 2	*T*	Cartesian vectors	–
PaINN^25^	1	1	1	≥ 2	*T*	Cartesian vectors	–
TorchMD-Net^28^	1	1	1	≥ 2	*T*	Cartesian vectors	–

The internal *l*_max_ specifies the angular information contained on the messaging function *M*_*t*_ indexed by the highest weights of the irreducible representations of *O*(3). The update *L*_max_ specifies the angular information in the update function. The total correlation order corresponds to the correlation order of the entire model as a function of individual atoms. The top nine models correspond to spherical equivariant interatomic potentials, whereas the bottom four models correspond to Cartesian equivariant interatomic potentials. –, not applicable.

The convolution of the SchNet network can be obtained by considering *T* ≥ 2, *ν* = 1, *L* = 0 and *l*_max_ = 0. The DimeNet invariant message-passing network includes higher-correlation-order messages (more precisely, three-body messages by incorporating angular information), meaning that *T* ≥ 2, *v* = 2, *L*_max_ = 0 and *l*_max_ = 5. NequIP corresponds to *T* ≥ 2, *ν* = 1, *L*_max_ ≥ 1 and *l*_max_ = *L*_max_, where the symmetrization of equation (4) can be simplified using ordinary Clebsch-Gordan coefficients, *C*:

6 $${m}_{i,kLM}^{(t)}=\sum _{{l}_{1}{m}_{1}{l}_{2}{m}_{2}}{C}_{{l}_{1}{m}_{1},{l}_{2}{m}_{2}}^{LM}\sum _{j\in {\mathcal{N}}(i)}{R}_{k{l}_{1}{l}_{2}L}^{(t)}({r}_{ji}){Y}_{{l}_{1}}^{\,{m}_{1}}({\hat{{\bf{r}}}}_{ji}){h}_{j,k{l}_{2}{m}_{2}}^{(t)}$$

The MACE model^35^ follows the Multi-ACE message of equation (4) and combines high local correlation order with equivariant messages in a spherical basis.

The models in the lower part of the table do not use a spherical harmonics expansion but work with Cartesian tensors. Nonetheless, they fit into this framework by considering the equivalence of vectors and *l* = 1 spherical tensors. The coordinate displacements present in EGNN^24^ and NewtonNet^26^, for example, can thus be rewritten as an *l* = 1 spherical expansion of the environment via a change of basis.

Based on the models presented in Table 1, the Multi-ACE framework lets us identify two main routes that have been taken thus far in building interatomic potentials. The models have either few layers and high local correlation order, like linear ACE (and other descriptor-based models), or many layers and low local correlation order, similar to NequIP.

### Message passing as a chemically inspired sparsification

One central aspect of message-passing models is the treatment of semi-local information: while in approaches such as ACE the atomic energy is only influenced by neighbouring atoms within the local cutoff sphere, the message-passing formalism iteratively propagates information, allowing semi-local information to be communicated. Equivariant MPNNs like NequIP update atom states on the basis of a tensor product between edge features and neighbouring atoms’ states, which leads to ‘chain-like’ information propagation.

Specifically, consider a much-simplified message-passing architecture with a single channel *k* and an update *U* that is just the identity:

7 $${h}_{i,LM}^{(t+1)}=\sum _{{l}_{1}{m}_{1}{l}_{2}{m}_{2}}{C}_{{l}_{1}{m}_{1},{l}_{2}{m}_{2}}^{LM}\sum _{j\in {\mathcal{N}}(i)}{R}_{{l}_{1}{l}_{2}L}^{(t)}({r}_{ji}){Y}_{{l}_{1}}^{\,{m}_{1}}({\hat{{\bf{r}}}}_{ji}){h}_{j,{l}_{2}{m}_{2}}^{(t)}.$$

We can write out the simple example of a two-layer update explicitly:

8 $$\begin{array}{lll}{h}_{i,LM}^{(2)}\\=\mathop{\sum}\limits _{{l}_{1}{m}_{1}{l}_{2}{m}_{2}}{C}_{{l}_{1}{m}_{1},{l}_{2}{m}_{2}}^{LM}\mathop{\sum}\limits _{{j}_{1}\in {\mathcal{N}}(i)}{R}_{{l}_{1}{l}_{2}L}^{(t)}({r}_{ji}){Y}_{{l}_{1}}^{\,{m}_{1}}({\hat{{\bf{r}}}}_{ji}){h}_{{j}_{1},{l}_{2}{m}_{2}}^{(1)}\\ =\,\mathop{\sum}\limits _{{l}_{1}{m}_{1}{l}_{2}{m}_{2}}{C}_{{l}_{1}{m}_{1},{l}_{2}{m}_{2}}^{LM}\mathop{\sum}\limits _{{j}_{1}\in {\mathcal{N}}(i)}{R}_{{l}_{1}{l}_{2}L}^{(t)}({r}_{ji}){Y}_{{l}_{1}}^{\,{m}_{1}}({\hat{{\bf{r}}}}_{ji})\\\quad\;\;\mathop{\sum}\limits _{{j}_{2}\in {\mathcal{N}}({j}_{1})}{R}_{{l}_{2}}^{(t)}({r}_{ji}){Y}_{{l}_{2}}^{\,{m}_{2}}({\hat{{\bf{r}}}}_{ji}){h}_{{j}_{2}}^{(0)}\end{array}$$

where we have assumed that ${h}_{{j}_{2}}^{(0)}$ is a scalar, learnable embedding of the chemical elements, such that it does not possess *l*.

This defines a pattern of information flow in which the state of *j*_2_ is first passed on to atom *j*_1_, resulting in the (*j*_2_,*j*_1_) correlation being captured. This is then passed on to atom *i*, which encodes the three-body interaction between atoms (*i*, *j*_1_,*j*_2_) on atom *i*. This scheme induces a chain-wise propagation mechanism (*j*_2_ → *j*_1_ → *i*), which is different from local models like ACE, in which the three-body correlation on atom *i* stems from an interaction between (*i*,*j*_1_) and (*i*,*j*_2_).

One can then, under the assumption of linearity, view equivariant MPNNs as a sparsification of an equivalent one-layer ACE model that instead has a larger cutoff radius *r*_cut,ACE_ = *T* × *r*_cut,MPNN_, where *r*_cut,ACE_ is the maximal distance of atoms that can see each other in a *T*-layer MPNN. While in a one-layer ACE, all clusters with central atom *i* would be considered, the MPNN formalism sparsifies this to only include walks along the graph (the topology of which is induced by local cutoffs) of length *T* that end on atom *i*.

**Fig. 3:**
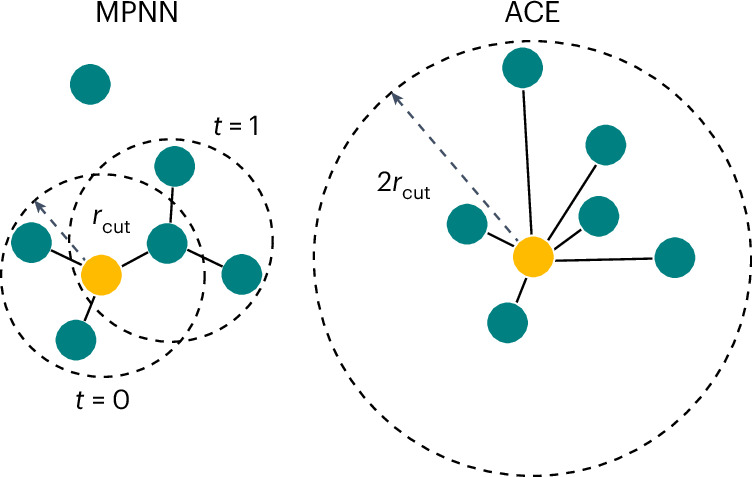
Receptive field of MPNNs. Comparison of the clusters formed by two iterations of message passing with cutoff *r*_cut_ at each iteration on the left for an MPNN and the clusters formed by ACE with cutoff 2*r*_cut_ on the right. In principle, both methods incorporate information from a distance of up to 2*r*_cut_, but in the case of the MPNN, only atoms that can be reached through a chain of closer intermediates contribute.

In practice, for typical settings of *T*, *r*_cut_ and *ν*, a local model like ACE with a cutoff of *T* × *r*_cut_ would be impractical due to the large number of atoms in the neighbourhood. Moreover, the clusters created by atom-centred representations for an equivalent cutoff to MPNNs are less physical, as illustrated in Fig. 3. Most physical interactions in chemistry are short-range and semi-local information propagates in a chain-like mechanism, thus making the message-passing sparsification correspond to the chemical bond topology. A more in-depth discussion on the relationship between message passing and semi-local information can be found in refs. ^33,34^.

## Choices in the equivariant interatomic potential design space

To render the Multi-ACE theory set out above of practical use, we analysed the design space of E(3)-equivariant interatomic potentials. We focused on two equivariant message-passing models: NequIP^23^ and its linearized body-order version, BOTNet^36^ (see the Supplementary Information for the precise architecture of BOTNet). We show how specific choices in the design space affect the performance of the models in terms of in-domain accuracy and smooth extrapolation and compare them with linear ACE, which is at a very different point of the framework.

### One-particle basis

The one-particle basis is at the core of any message-passing interatomic potential (Methods). In the most general case, the one-particle basis is denoted ${\phi }_{kvL}^{(t)}({\sigma }_{i}^{(t)},{\sigma }_{j}^{(t)})$ as introduced in equation (2). Below, we analyse some of the choices that can be made regarding the treatment of the chemical elements via the ${T}_{kcL}^{\,(t)}({{{h}}}_{j}^{(t)},{{{\theta }}}_{{{i}}},{{{\theta }}}_{{{j}}})$ functions and the treatment of ${R}_{kc{l}_{1}L}^{(t)}({r}_{ji})$.

#### Treatment of the chemical elements

The continuous embedding used in MPNNs is analogous to having many separate linear ACEs that are all sensitive to the chemical elements in a different learnable way. After each message-passing step, the chemical element channels are mixed via a learnable transformation. It is interesting to note that the chemical identity of the neighbouring atom (the sender) only enters directly at iteration *t* = 0 when ${{{h}}}_{{{j}}}^{(0)}$ is the one-hot encoding of the chemical elements; after this it is only indirectly dependent on the sender element via the output of the previous layer.

In this section we analyse the effect of increasing *k*, which corresponds to the dimension of the chemical element embedding. Table 2 compares NequIP models with increasing *k*. The number of uncoupled (chemical) channels substantially affects the number of parameters. However, the scaling is nearly linear with the number of channels, rather than a power law (equal to the correlation order) with the number of different elements, which would be the case if a discrete chemical element index and the complete linear ACE basis were used. The formal relationship between the fully coupled and uncoupled treatments of the chemical elements is described in ref. ^37^. It is also interesting to note that, as is typical in deep learning, over-parameterized models often achieve better results^38^ not only in-domain (at low temperature) but also when extrapolating out-of-domain (at high temperature).

**Table 2:** Root-mean-square error on the 3BPA dataset with NequIP networks of different chemical embedding size

*N*_channels_		16	32	64	128
Number of parameters		437,336	1,130,648	3,415,832	11,580,440
300 K	*E* (meV)	3.7	3.1	3.0 (0.2)	**2.9**
	*F* (meV Å^−1^)	12.9	11.9	11.6 (0.2)	**10.6**
600 K	*E* (meV)	12.9	12.7	11.9 (1.1)	**10.7**
	*F* (meV Å^−1^)	32.1	30.3	29.4 (0.8)	**26.9**
1,200 K	*E* (meV)	48.6	49.5	49.8 (4.0)	**46.0**
	*F* (meV Å^−1^)	104.2	101.6	97.1 (5.6)	**86.6**

Energy (*E*) and force (*F*) errors are shown for NequIP networks of increasing feature vector size, trained and tested on configurations of the flexible drug-like molecule 3-(benzyloxy)pyridin-2-amine (3BPA) at the temperatures indicated. All models were trained at 300 K. All results were generated with the nequip code base. Bold numbers correspond to lowest error in each row. Three models were trained (using different random seeds to initialise the weights) with 64 channels, and the standard deviation of the errors are shown in parentheses in the corresponding column.

A further advantage of the element embedding approach is that it allows some alchemical learning. The embeddings can learn a latent representation of the chemical elements and provide meaningful predictions for combinations of elements that do not appear simultaneously in the training set^39^. A demonstration of this alchemical learning is also presented in the Supplementary Information to compare NequIP and BOTNet to the non-element-embedded linear ACE. The experiment shows how the element embedding leads to physically sensible dimer curves, even for interactions that are not present in the training set.

#### Radial basis

There is considerable freedom in choosing a functional form for ${R}_{kc{l}_{1}L}^{(t)}({r}_{ji})$. In the context of atom-density-based environment representations such as SOAP^3^, ACSF^2^ and the bispectrum (SNAP)^40^, the importance of the radial basis has been long known, and many strategies have been developed to improve it. Adopting the best radial basis has been a continuous source of improvement for models in the past. For example, in the case of SOAP, improving the radial basis led to models that were more efficient, smoother and faster^41–45^.

The most straightforward choice for a radial basis, used (for example) by linear ACE, is a set of fixed orthogonal polynomial basis functions that are the same for each chemical element and do not depend on *l*. The dependence on the atom types only enters via the distance transform^12,46^. This distance transform scales the interatomic distances to be in the domain of the orthogonal radial basis. Its form can be dependent on the chemical elements of the two atoms, accounting for the differences in atomic radii.

Recently, much work has shown that it can be advantageous to optimize the radial basis in a data-driven way. This can be done a priori^47^ or can be optimized during the training of the model^48^.

NequIP also uses a learnable radial basis that is dependent on the tuple (*k*,*l*_1_,*l*_2_,*L*), where *l*_1_ corresponds to the representation of the spherical harmonics ${Y}_{{l}_{1}}^{\,{m}_{1}}({\hat{{\bf{r}}}}_{ji})$, *l*_2_ to the representation of the equivariant message ${h}_{j,{l}_{2}{m}_{2}}^{(t)}$ and there is a different radial basis for each *L*:

9 $${R}_{k{l}_{1}{l}_{2}L}^{(t)}({r}_{ji})=\,\text{MLP}\,\left({R}_{n}({r}_{ji}){f}_{{\rm{cut}}}({r}_{ji})\right),$$

where MLP is a multi-layer perceptron. Typically, the number of layers used in this MLP is three. *R*_*n*_ are a set of Bessel basis polynomials and *f*_cut_(*r*_*ji*_) is a cutoff function such that $\mathop{\lim }\nolimits_{{r}_{ji}\to 0}{f}_{{\rm{cut}}}({r}_{ji})=0$ but orthogonality of the different basis functions is not enforced. This type of radial basis allows improved flexibility in spatial resolution when combining features of different symmetries. We refer to this radial basis as the element-agnostic radial basis, as it is independent of the chemical elements.

BOTNet uses a similar learnable radial basis, but it is also dependent on the sender atom chemical element. This is achieved by forming radial basis functions with the *k* multi-index running over *N*_channels_ = *N*_embedding_ × *N*_elements_. This means that BOTNet will have a separate radial basis in each chemical embedding channel for each neighbour chemical element and that the *T*_*kc*_ function will pick up the appropriate one via its dependence on *θ*_*j*_ (see equation (2)). This radial basis can be written as:

10 $${R}_{k{l}_{1}{l}_{2}L}^{(t)}({r}_{ji})=\sum _{n}{W}_{kn({l}_{1}{l}_{2}L)}^{\,(t)}{R}_{n}({r}_{ji}){f}_{{\rm{cut}}}({r}_{ji}),$$

where ${W}_{kn({l}_{1}{l}_{2}L)}^{\,(t)}$ is an array of weights of dimensions [*N*_channels_, *N*_basis_, *N*_paths_], *N*_basis_ is the number of Bessel basis functions *R*_*n*_ and *N*_paths_ is the number of combination of products of a given symmetry between the equivariant feature ${h}_{j,{l}_{2}{m}_{2}}$ and the spherical harmonics. We refer to this type of radial basis as the element-dependent radial basis because it explicitly depends on the chemical element of atom *j* via the weight array.

We have observed that the element-dependent radial basis gives better training and validation accuracy. However, for extreme extrapolation (such as with bond breaking), we have found that the agnostic radial basis is a better choice, particularly with the correct normalization (as discussed in the Supplementary Information).

### Nonlinear activations

The body ordering, as defined in the Methods, is a central property of classical force fields and has proved to be a very successful approximation of quantum mechanical systems^8^. The linear version of ACE is body ordered by construction, but most other machine learning approaches do not have this structure. The use of body-ordered models was thought to be beneficial because it enforces the learning of low-dimensional representations of the data, which is an excellent inductive bias for better extrapolation. In the following, we analyse different nonlinear activations and their effects on body ordering.

The ACE message-passing equation in equation (4) is a nonlinear operation and is fundamentally related to the tensor product of the *O*(3) group. The effect of this tensor-product nonlinearity is to increase the body order of each layer by *ν*. Most previously published MPNN architectures have *ν* = 1. Beyond the tensor product, it is possible to include other types of nonlinearity in *U*_*t*_ of equation (1) by taking ${U}_{t}=\left({\sigma }_{i}^{(t)},{{{m}}}_{i}^{(t)}\right)={\mathcal{F}}\left({W}^{\,(t)}{{{m}}}_{i}^{(t)}\right)$ where ${\mathcal{F}}$ is a generic nonlinear function and *W* ^(*t*)^ is an [*N*_channels_ × *N*_channels_ × (*L*_max_ + 1)] learnable weight matrix that linearly mixes *k*. It is important to note that ${\mathcal{F}}$ does not preserve equivariance when applied to equivariant features. A common strategy is to use gated equivariant nonlinearities, which are summarized in the Supplementary Information. In the following, when we compare nonlinearities the models differ only in the choice of nonlinearities applied to the invariant parts of the models; the equivariant nonlinearities are always kept the same.

If the model is explicitly body-ordered and equivariant, only a smaller subset of nonlinearities that preserve the equivariance can then be used. The central remark is that a nonlinearity preserves body ordering if it admits a finite Taylor expansion. A detailed example showing how the Sigmoid Linear Unit (SiLU) nonlinearity destroys the body-ordered structure is presented in the Supplementary Information. Two types of nonlinearity preserve the body-ordered structure; the first is known as the kernel trick and consists of using nonlinearities with a finite Taylor expansion (such as the squared norm) to raise the body order of the representation^3^.

The approach taken in designing BOTNet was to create a body-ordered model during the first five message-passing layers by removing all nonlinear activations from the update but making the last readout nonlinear with an infinite body order. In this way, the last readout function is responsible for representing the residual of the body-order expansion not captured by the first five layers. This energy decomposition enforces the learning of low-dimensional structures because the low-body-order part of the energy appears explicitly. The corresponding energy expansion of BOTNet is:

11 $$E={E}^{\,(0)}+\mathop{\sum }\limits_{i=1}^{N}{E}_{i}^{\,(0)}({{{\bf{r}}}}_{i})+\mathop{\sum }\limits_{1\le i < j\le N}^{N}{E}_{i,\,j}^{\,(1)}({{{{\bf{r}}}}}_{i},{{{\bf{r}}}}_{j})+\cdots +{E}_{{\rm{rest}}},$$

where ${E}_{{\rm{rest}}}={\mathcal{F}}({{{m}}}^{(T\,)}({{{\bf{r}}}}_{{i}_{1}},\ldots ,{{{\bf{r}}}}_{{i}_{n}}))$ is a general nonlinear term that accounts for all of the missing contributions not captured by the previous body-ordered layers.

Models that use different nonlinearities are compared in Table 3. It is clear that in the case of NequIP, the choice of nonlinearity is crucial; using tanh instead of SiLU makes the results significantly worse. This is probably because the tanh function has 0 gradient for large positive and negative inputs, which makes the optimization difficult due to vanishing gradients^49^. This makes models with tanh nonlinearity even worse than not using any nonlinearities at all (other than the tensor product). In the case of BOTNet, we can see that adding a nonlinear layer to a strictly body-ordered model to account for the higher-order terms in the truncated body-ordered expansion significantly improves the results. The normalization row indicates the type of data normalization used for the experiments (for further information on normalization, see the Supplementary Information).

**Table 3:** Root-mean-square energy and force errors on the 3BPA dataset for different choices of nonlinear and linear models

Model		NequIP tanh	NequIP Silu	NequIP linear	BOTNet linear	BOTNet
Code		botnet	nequip	botnet	botnet	botnet
300 K	*E* (meV)	4.8	**3.0** (0.2)	3.7	3.3	3.1 (0.13)
	*F* (meV Å^−1^)	18.5	11.6 (0.2)	13.9	12.0	**11.0** (0.14)
600 K	*E* (meV)	20.1	11.9 (1.1)	15.4	11.8	**11.5** (0.6)
	*F* (meV Å^−1^)	42.5	29.4 (0.8)	34.1	30.0	**26.7** (0.29)
1,200 K	*E* (meV)	75.7	49.8 (4.0)	61.92	53.7	**39.1** (1.1)
	*F* (meV Å^−1^)	156.1	97.1 (5.6)	109.5	97.8	**81.1** (1.5)

Models in the first two columns of data use $\lambda =\sqrt{{\left\langle \#{\mathcal{N}}(k)\right\rangle }_{k}}$ and the models in the remaining columns use $\lambda ={\left\langle \#{\mathcal{N}}(k)\right\rangle }_{k}$ (Supplementary Information). Linear models refer to models without any nonlinear activation. Bold numbers correspond to lowest error in each row.

## Discussion

In this Article we have introduced Multi-ACE, a framework in which many previously published E(3)-equivariant (or invariant) machine learning interatomic potentials can be understood. Using this framework, we have identified a large design space and have systematically studied how different choices made in the different models affect the accuracy, smoothness and extrapolation of the fitted interatomic potentials. In the Supplementary Information we show the performance of the equivariant graph neural network models in a broader context, comparing them with earlier approaches.

We used NequIP as an example to probe each of the design choices and created the BOTNet model, which retains the most crucial elements of NequIP (the equivariant tensor product and the learnable residual architecture) but makes different choices for the radial basis, the use of nonlinear activations and readouts, making it an explicitly body-ordered MPNN model. Our study also highlights the crucial importance of internal normalization and the effect of data normalization on both accuracy and extrapolation. One particularly interesting region of the design space relates to the use of locally many-body features in a message-passing model, which has been the subject of investigation in some studies^34,35^.

## Methods

### Equivariant ACE with continuous embedding and uncoupled channels

ACE^7,8^ was originally proposed as a framework for deriving an efficient body-ordered symmetric polynomial basis to represent functions of atomic neighbourhoods. It has been shown that many of the previously proposed symmetrized atomic field representations^1^, such as ACSF^2^, SOAP^3^, moment tensor potential basis functions^10^ and the hyperspherical bispectrum^9^ used by SNAP^40^, can be expressed in terms of the ACE basis^7,8,11,50^.

In the following we present a version of the ACE formalism for deriving E(3)-invariant and equivariant basis functions that incorporates a continuous embedding of chemical elements and will serve as an important building block of the Multi-ACE framework.

#### One-particle basis

The first step in constructing the ACE framework is to define the one-particle basis, which is used to describe the spatial arrangement of atoms *j* around the atom *i*:

12 $${\phi }_{nlm{z}_{i}{z}_{j}}({{{r}}}_{ji})={R}_{nl{z}_{i}{z}_{j}}({r}_{ji}){Y}_{l}^{\,m}({\hat{{\bf{r}}}}_{ji}),$$

where the indices *z*_*i*_ and *z*_*j*_ refer to the chemical elements of atoms *i* and *j*. The one-particle basis functions are formed as the product of a set of orthogonal radial basis functions *R*_*nl*_ and spherical harmonics ${Y}_{l}^{\,m}$. The positional argument *r*_*ji*_ in equation (12) can be obtained from $({\sigma }_{i}^{(t)},{\sigma }_{j}^{(t)})$, meaning that the value of the one-particle basis function depends on the states of two atoms.

The formulation in equation (12) uses discrete chemical element labels. The drawback of this approach is that the number of different basis functions rapidly increases with the number of chemical elements in the system. Given *S* different chemical elements and maximum body order *N*, the number of basis functions is proportional to *S*^*N*^. By contrast, MPNNs typically leverage a learnable mapping from the discrete chemical element labels to a continuous fixed-length representation. Using such an embedding with ACE eliminates the scaling of the number of basis functions with the number of chemical elements. The one-particle basis can be generalized to allow this continuous embedding via a set of functions whose two indices we explain below:

13 $${\phi }_{kv}({\sigma }_{i},{\sigma }_{j})={R}_{kcl}({r}_{ji}){Y}_{l}^{\,m}({\hat{{\bf{r}}}}_{ji}){T}_{kc}({{{\theta }}}_{i},{{{\theta }}}_{j}),$$

where *T*_*kc*_ is a generic function of the chemical attributes *θ*_*i*_ and *θ*_*j*_ and is endowed with two indices, *k* and *c*, and the radial basis likewise. Of these, *c*, together with *l* and *m*, will be coupled together when we form many-body basis functions (see equation (15)). These coupled indices are collected into a single multi-index (*v* ≡ *lmc*) for ease of notation. We refer to *k* as the uncoupled index.

Beyond the chemical element labels, *T*_*kc*_ can account for the dependence of the one-particle basis functions on other attributes of the atoms, such as the charge, magnetic moment^11^ or learnable features. Furthermore, the output of *T*_*kc*_ can be invariant or equivariant to rotations. In the case of equivariant outputs, *k* (in the uncoupled case) or *c* (in the coupled case) will themselves be multi-indices that contain additional indices (for example, *l*′ and *m*′) that describe the transformation properties of these outputs.

To recover equation (12) with the discrete element labels, we set *θ*_*i*_,*θ*_*j*_ to *z*_*i*_,*z*_*j*_ and assume that *k* ≡ 1 (that is, there are no uncoupled indices). Furthermore, we set *c* to be a multi-index (*c* ≡ *nz*_*i*_*z*_*j*_) with *T*_*kc*_ being an index selector ${T}_{kc}={T}_{1n{z}_{i}{z}_{j}}={\delta }_{{z}_{i}{\theta }_{i}}{\delta }_{{z}_{j}{\theta }_{j}}$. In this case, the index *n* of ${R}_{nl{z}_{i}{z}_{j}}$ in equation (12) is also part of *c*.

In the language of MPNNs, the values of the one-particle basis functions would be thought of as edge features of a graph neural network model. This graph would be directed, as the one-particle basis functions are not symmetric with respect to the swapping of the central atom *i* and the neighbouring atom *j*.

#### Higher-order basis functions

A key innovation of ACE was the construction of a complete many-body basis, which can be computed at a constant cost per basis function^51^. The high-body-order features can be computed without having to explicitly sum over all triplets, quadruplets and so on, which is achieved by what came to be called the density trick^1^, introduced originally for the fast evaluation of high-body-order descriptors^3,9^. This allows any E(3)-equivariant function of an atomic neighbourhood to be expanded using a systematic body-ordered expansion at a low computational cost^8^.

The next step of the ACE construction is analogous to traditional message passing: we sum the values of the one-particle basis functions evaluated on the neighbours to form the atomic or *A* basis. This corresponds to a projection of the one-particle basis on the atomic density. Therefore, in the atomic environment representation literature, this step is often referred to as the density projection^1^:

14 $${A}_{i,kv}=\sum _{j\in {\mathcal{N}}(i)}{\phi }_{kv}({\sigma }_{i},{\sigma }_{j}).$$

The *A* basis is invariant with respect to the permutations of the neighbouring atoms, and its elements are two-body functions in the sense of the definition in equation (27). This means that this basis can represent functions that depend on all neighbours’ positions but can be decomposed into a sum of two-body terms.

Then, to create basis functions with higher body order, we form products of the *A* basis functions to obtain the product basis, *A*_*i*,*kv*_:

15 $$\begin{array}{c}{{{A}}}_{i,k\,{\bf{v}}}=\mathop{\prod }\limits_{\xi =1}^{\nu }{A}_{i,k{v}_{\xi }},\quad {\bf{v}}=({v}_{1},\ldots ,{v}_{\nu }),\end{array}$$

where *ν* denotes the correlation order and the array index **v** collects the multi-indices of the individual *A*-basis functions, representing a *ν* tuple. The product basis is a complete basis of permutation-invariant functions of the atomic environment.

Taking the product of *νA* basis functions results in basis functions of correlation order *ν*, which thus have body order *ν* + 1 due to the central atom. In the language of density-based representations, these tensor products correspond to *ν* correlations of the density of atoms in the atomic neighbourhood^33^.

For example, the *ν* = 3, four-body basis functions have the form:

16 $${{{A}}}_{i,k\,{\bf{v}}}={A}_{i,k{v}_{1}}{A}_{i,k{v}_{2}}{A}_{i,k{v}_{3}},$$

where **v** = (*v*_1_*v*_2_*v*_3_). This illustrates the difference between the uncoupled *k* channels (dimension) and the coupled *v* channels: we did not form products with respect to the indices collected in *k*. Note that in linear ACE, as described in refs. ^7,8,12^, the tensor product is taken with respect to all of the indices (radial, angular and chemical elements) in **v**, and no uncoupled indices are used.

#### Symmetrization of basis functions

The product basis constructed in the previous section linearly spans the space of permutationally and translationally invariant functions but does not account for rotational invariance or equivariance of the predicted properties or intermediate features. To create rotationally invariant or equivariant basis functions, the product basis must be symmetrized with respect to *O*(3). The symmetrization takes its most general form as an averaging over all possible rotations of the neighbourhood. In the case of rotationally invariant basis functions, this averaging is expressed as an integral of the product basis over rotated local environments:

17 $${B}_{{i,k}\,{{v}}}:= {\int}_{O(3)}{{{A}}}_{i,k{\bf{v}}}\left({\left\{Q\cdot \left({\sigma }_{i},{\sigma }_{j}\right)\right\}}_{j\in {\mathcal{N}}(i)}\right)\,{\mathrm{d}}Q,$$

where we explicitly define the dependence of the product basis on the atomic states, and *Q* ⋅ (*σ*_*i*_,*σ*_*j*_) = (*Q* ⋅ *σ*_*i*_,*Q* ⋅ *σ*_*j*_) denotes the action of the rotation on a pair of atomic states. The above integral is purely formal. To explicitly create a spanning set of the symmetric *B* functions above, one can instead use tensor contractions as the angular dependence of the product basis is expressed using products of spherical harmonics (see equation (20) below).

The construction of equation (17) is readily generalized if equivariant features are required^11,52,53^. If the action of a rotation *Q* on a feature *h* is represented by a matrix *D*(*Q*), then we can write the equivariance constraint as:

18 $${{D}}{(Q)}^{-1}{{h}}\left({\left\{Q\cdot \left({\sigma }_{i},{\sigma }_{j}\right)\right\}}_{j\in {\mathcal{N}}(i)}\right)={{h}}\left({\left\{{\sigma }_{i},{\sigma }_{j}\right\}}_{j\in {\mathcal{N}}(i)}\right).$$

To linearly expand *h*, the basis functions must satisfy the same symmetries. This is achieved by defining the symmetrized basis as:

19 $${B}_{{i,k}\,{{v},\alpha }}={\int}_{O(3)}({{D}}{(Q)}^{-1}{e}_{\alpha }){{{A}}}_{{i,k}\,{{v}}}\left({\left\{Q\cdot \left({\sigma }_{i},{\sigma }_{j}\right)\right\}}_{j\in {\mathcal{N}}(i)}\right)\,{\mathrm{d}}Q,$$

where *e*_*α*_ is a basis of the feature space *h*. This approach can be applied to parameterize tensors of any order, both in Cartesian and spherical coordinates. For instance, if *h* represents a Euclidean three vector, *e*_*α*_ can just be the three Cartesian unit vectors, $\hat{{\bf{x}}}$, $\hat{{\bf{y}}}$ and $\hat{{\bf{z}}}$.

From here we focus on features with spherical *L* equivariance and label them *h*_*L*_ accordingly, with the corresponding basis functions denoted *B*_*i*,*kαLM*_. The matrices *D*(*Q*) become the Wigner-*D* matrices; that is, *D*^*L*^(*Q*).

The integration over the rotations can be reduced to recursions of products of Wigner-*D* matrices and carried out explicitly as a tensor contraction^8,53^. It is then possible to create a spanning set of *L*-equivariant features of the integrals of the types of equations (17) and (19) using linear operations. This can be done by introducing the generalized coupling coefficients:

20 $${B}_{i,k\eta ,LM}=\sum _{{\bf{v}}}{{\mathcal{C}}}_{\eta ,{\bf{v}}}^{LM}{{{A}}}_{i,k\,\bf{v}},$$

where the output index *η* enumerates the different possible combinations of *A*_*i*,*k***v**_ that have *L*. For a detailed discussion of the invariant case, see ref. ^8^.

Using spherical coordinates for the features, ${{\mathcal{C}}}_{\eta ,{\bf{v}}}^{LM}$ corresponds to the generalized Clebsch–Gordan coefficients and *L* corresponds to the usual labelling of the *O*(3) irreducible representations. An additional degree of freedom can be introduced by having a different *A*_*i*,*k***v***L*_ product basis for each *L* (for example, by choosing different one-particle basis functions depending on *L*). This is a choice made for NequIP. The creation of symmetric high-body-order basis functions is summarized in Fig. 1.

The functions *B*_*i*,*kη*,*LM*_ form a spanning set, meaning that all (*ν* + 1)-body functions with symmetry *L* of the atomic environment can be represented as a linear combination of *B* functions^8^. The values of the *B* functions can be combined into an output *m*_*i*,*kLM*_ on each atom *i* and each channel *k* via a learnable linear transformation:

21 $${m}_{i,kLM}=\sum _{\eta }{w}_{k\eta L}{B}_{i,k\eta ,LM}.$$

Finally, to generate the target output for atom *i*, *k* can be mixed via a learnable (linear or nonlinear) function ${{{\varPhi }}}_{i,L}={\mathcal{F}}({{{m}}}_{i,L})$.

### MPNN potentials

In this section we summarize the MPNN framework^17,54,55^ for fitting interatomic potentials and use this framework to elucidate the connections between linear ACE and MPNNs. The comparison of a wide range of models within this framework helps to identify and explain their key similarities and differences.

MPNNs are a class of graph neural networks that can parameterize a mapping from the space of labelled graphs to a vector space of features. They can be used to parameterize interatomic potentials by making atoms correspond to the nodes of the graph, and an edge connects two nodes if their distance is less than *r*_cut_. The model maps a set of atoms with element types positioned in the three-dimensional Euclidean space to the total potential energy. Typically, *r*_cut_ is several times larger than the length of a covalent bond. Thus, the corresponding graph is quite different from the typically drawn bonding graph of a molecule; instead, it represents the spatial relationships between atoms on a larger length scale. We denote the set of neighbours of an atom *i* (that is, atoms within the cutoff distance) ${\mathcal{N}}(i)$.

### Semi-local states

We denote the state of an atom *i* as the tuple ${\sigma }_{i}^{(t)}$:

22 $${\sigma }_{i}^{(t)}=\left({{\bf{r}}}_{i},{{{\theta }}}_{i},{{{h}}}_{i}^{(t)}\right),$$

where **r**_*i*_ denotes the atom’s Cartesian position vector, *θ*_*i*_ a set of its fixed attributes such as the chemical element (typically represented by a one-hot encoding), and ${{{h}}}_{i}^{(t)}$ its learnable features. These features, unlike the attributes, are updated after each message-passing iteration *t* on the basis of the states of the atoms connected to atom *i*. We refer to the states as semi-local, as the features will ultimately depend on the states of atoms far away (around 10 to 40 Å, depending on the local neighbourhood cutoff and the number of iterations). A smooth cutoff mechanism is employed such that the updates are continuous when atoms leave or enter each other’s local neighbourhood.

### Message-passing formalism

We reformulate the original MPNN equations^17^ for atomic states. In general, an MPNN potential consists of a message-passing phase and a readout phase. In the message-passing phase, ${{{h}}}_{i}^{(t)}$ are updated based on ${{{m}}}_{i}^{(t)}$ derived from the states of the neighbouring atoms within the set ${\mathcal{N}}(i)$:

23 $${{{m}}}_{i}^{(t)}=\frac{1}{\lambda }\bigoplus _{j\in {\mathcal{N}}(i)}{M}_{t}\left({\sigma }_{i}^{(t)},{\sigma }_{j}^{(t)}\right),$$

where ${\bigoplus }_{j\in {\mathcal{N}}(i)}$ refers to a permutation-invariant pooling operation over the neighbours of atom *i*, and *λ* can correspond to, for example, the average number of neighbours across the training set (see the Supplementary Information for a detailed analysis of the role of this normalization). *M*_*t*_ denotes a learnable function acting on the states of atoms *i* and *j*. The most widely used permutation-invariant pooling operation is the sum over the neighbours. This operation creates ${{{m}}}_{i}^{(t)}$, which are two-body in nature—that is, linear combinations of functions that simultaneously depend on the features of only two atoms. Then ${{{m}}}_{i}^{(t)}$ can be combined with the features of atom *i* by a learnable update function, *U*_*t*_:

24 $${\sigma }_{i}^{(t+1)}\equiv \left({{\bf{r}}}_{i},{{{\theta }}}_{i},{{{h}}}_{i}^{(t+1)}\right)=\left({{\bf{r}}}_{i},{{{\theta }}}_{i},{U}_{t}\left({\sigma }_{i}^{(t)},{{{m}}}_{i}^{(t)}\right)\right).$$

In *U*_*t*_, it is possible to form higher-body-order messages by (for example) applying a square function to the message to obtain a linear combination of three-body functions that simultaneously depend on the central atom and two of its neighbours. Both *M*_*t*_ and *U*_*t*_, depend on the iteration index *t*.

In the readout phase, learnable functions ${{\mathcal{R}}}_{t}$ map the atomic states onto atomic site energies:

25 $${E}_{i}=\sum _{t}{{\mathcal{R}}}_{t}\left({\sigma }_{i}^{(t)}\right).$$

At this point, some models use the atomic states from every iteration, while others use only a single readout function that takes the state after the final iteration and maps it to the site energy.

### Equivariant messages

Physical properties, such as the energy or the dipole moment, transform in specific ways under the action of certain symmetry operations, such as translations and rotations of the atomic coordinates. For atomistic modelling, the E(3) symmetry group is of special interest. For example, if a molecule is rotated in space, the predicted dipole moment should rotate accordingly, whereas the energy should remain unchanged. Here we restricted ourselves to rotational and reflectional symmetries, the *O*(3) group, as translation invariance can be ensured by working with interatomic displacement vectors **r**_*ji*_ ≔ **r**_*j*_ − **r**_*i*_.

A straightforward and convenient way to ensure that the outputs of models transform correctly is to impose constraints on the internal representations of the model to respect these symmetries. We categorize the features of an equivariant neural network based on how they transform under the symmetry operations of the inputs. Formally we can think of ${{{m}}}_{i,L}^{(t)}$ as a function of the input positions **r**_*i*_ (here the dependence on attributes *θ*_*i*_ is suppressed for the sake of brevity). Then we say that ${{{m}}}_{i,L}^{(t)}$ is rotationally equivariant (with *L*) if it transforms according to the irreducible representation *L* of the symmetry group:

26 $$\begin{array}{l}{{{m}}}_{i,L}^{(t)}\left(Q\cdot ({{\bf{r}}}_{1},\ldots ,{{\bf{r}}}_{N})\right)={{{D}}}^{L}(Q){{{m}}}_{i,L}^{(t)}({{\bf{r}}}_{1},\ldots ,{{\bf{r}}}_{N}),\\ \forall Q\in \,\text{O}\,(3)\end{array}$$

where *Q* ⋅ (**r**_1_, …, **r**_*N*_) denotes the action of an arbitrary rotation matrix *Q* on the set of atomic positions (**r**_1_, …, **r**_*N*_) and *D*^*L*^(*Q*) is the corresponding Wigner-D matrix, the irreducible representations of the *O*(3) group^56^. Hence, a message indexed by *L* transforms like the spherical harmonic ${Y}_{L}^{M}$ under rotation.

An important practical choice for implementing equivariant neural networks is the basis in which features and messages are expressed. For the rest of this Article, we will assume that they are encoded in spherical coordinates. This is in line with many equivariant models such as SOAP-GAP^3^, SNAP^40^, ACE^7,11^ and its recursive implementations such as ref. ^50^, NICE^53^, NequIP^23^, equivariant transformer^28^ and SEGNNs^29^. By contrast, some equivariant MPNNs (such as NewtonNet^26^, EGNN^24^ or PaINN^25^) express the features in Cartesian coordinates. Given that models in this latter class use Euclidean vectors, which correspond to *L* = 1 spherical vectors, they fit into the same framework through a change of basis. Spherical vectors transform according to *D*_1_(*Q*), which correspond to 3 × 3 rotation matrices. Some models, such as SchNet^16^ and DimeNet^19^, employ only invariant messages (that is, *L* = 0 equivariance).

### Body-ordered messages

The body-order expansion of a general multivariate function is

27 $$\begin{array}{rcl}F\left({\{{r}_{i}\}}_{i = 1}^{N}\right)&=&{f}^{\,(0)}+\mathop{\sum }\limits_{i=1}^{N}{f}^{\,(1)}({r}_{i})+\mathop{\sum}\limits _{1\le i < j\le N}{f}^{\,(2)}({r}_{i},{r}_{j})\\ &&\cdots +\mathop{\sum}\limits _{1\le {i}_{1} < \ldots < {i}_{N}\le N}{f}^{\,(N)}({r}_{({i}_{1})},{r}_{({i}_{2})},\ldots ,{r}_{({i}_{N})}).\end{array}$$

If the magnitude of higher-order terms is sufficiently small that they can be truncated, this expansion can be a powerful tool for approximating high-dimensional functions. The concept of body ordering also appears in quantum mechanics^57,58^, and there is ample empirical evidence that a body-ordered expansion of the potential energy converges rapidly for many systems^8^.

By explicitly controlling the body order, one can efficiently learn low-dimensional representations corresponding to low-body-order terms. This is suggested to lead to interatomic potentials with enhanced generalization ability^59^. For a message *m*^(*t*)^, the body order can be defined as the largest integer ${\mathcal{T}}$ such that:

28 $$\frac{{\partial }^{{\mathcal{T}}}{{\boldsymbol{m}}}^{(t)}}{\partial {r}_{{i}_{1}}\ldots \partial {r}_{{i}_{{\mathcal{T}}}}}\ne 0,\quad \forall \,\,\text{distinct}\,\,({i}_{1},\ldots ,{i}_{{\mathcal{T}}})$$

holds, where the elements in the tuple $({i}_{1},\ldots ,{i}_{{\mathcal{T}}})$ are all distinct, and for all $\tau > {\mathcal{T}}$ the left-hand side of equation (28) is identically zero^8,60^.

We call a model body-ordered if it can be written explicitly in the form of equation (27) with all terms up to ${\mathcal{T}}$ present. This is in contrast to non-body-ordered models, in which either the expansion is infinite or only a subset of terms is present. To achieve body ordering in an MPNN model one needs linear update and readout functions. This is because nonlinear activation functions, such as the hyperbolic tangent function (tanh), the exponential function and vector normalizations, have infinite Taylor-series expansions, which make the body order infinite without all the terms in equation (27) being explicitly present.

## Supplementary information

Supplementary Information Supplementary benchmark results, Supplementary Figs. 1–7, Tables 1–6 and text.

## Data Availability

The datasets used in the computational experiments are described in the Supplementary Information and are available via Zenodo at 10.5281/zenodo.14013500 (ref. ^61^).

## Extended data

**Extended Data Table 1:** Different machine learning potentials in the framework of MPNNs

	SchNet	NequIP	Linear ACE
Message function *M*_*t*_	${R}_{k}^{(t)}\left(| | {r}_{j}-{r}_{i}| | \right){h}_{j,k}^{(t)}$	${R}_{k{l}_{1}{l}_{2}L}^{(t)}({r}_{ji}){Y}_{{l}_{1}}^{{m}_{1}}({{\hat{\boldsymbol{r}}}}_{{\boldsymbol{ji}}}){h}_{j,k{l}_{2}{m}_{2}}^{(t)}$	${R}_{n}({r}_{ji}){Y}_{l}^{m}({\hat{{\boldsymbol{r}}}}_{ji}){\delta }_{{z}_{i}{\theta }_{i}}{\delta }_{{z}_{j}{\theta }_{j}}$
Symmetric pooling ${\bigoplus }_{j\in {\mathcal{N}}(i)}$	${\sum }_{j\in {\mathcal{N}}(i)}$	${\sum }_{{l}_{1}{m}_{1}{l}_{2}{m}_{2}}{{\mathcal{C}}}_{{l}_{1}{m}_{1}{l}_{2}{m}_{2}}^{LM}{\sum }_{j\in {\mathcal{N}}(i)}$	${\sum }_{\eta }{w}_{\eta }\,{\sum }_{{\boldsymbol{v}}}{{\mathcal{C}}}_{\eta ,{\boldsymbol{v}}}^{00}\mathop{\prod }\nolimits_{\xi = 1}^{\nu }{\sum }_{j\in {\mathcal{N}}(i)}$
Update function *U*_*t*_	${{\boldsymbol{h}}}_{i}^{(t)}+\tanh \left({W}^{(t)}{{\boldsymbol{m}}}_{i}^{(t)}+{{\boldsymbol{b}}}^{(t)}\right)$	${{\boldsymbol{h}}}_{i}^{(t)}+\tanh \left(\parallel {W}^{(t)}{{\boldsymbol{m}}}_{i}^{(t)}{\parallel }^{2}\right){W}^{(t)}{{\boldsymbol{m}}}_{i}^{(t)}$	–

We identify SchNet, NequIP, and ACE as examples of MPNNs and exhibit their explicit components in the design space: the message, symmetric pooling, and update functions. Note that in NequIP, the choice of nonlinearity is not fixed, and we have chosen a normed activation with $\tanh$ to be shown here. In each case, learnable parameters (weights) are shown as *W* and biases as *b*.

## Code availability

Two different codes were used to conduct the experiments. All experiments labelled ‘nequip’ in Table 3 were conducted with NequIP (github.com/mir-group/nequip) version 0.5.4, which is available via Zenodo^62^, in conjunction with version 0.4.4 of the e3nn library^63^ and PyTorch version 1.10.0 (ref. ^64^). All experiments labelled ‘botnet’ in Table 3 were conducted with BOTNet^65^, which is available via GitHub at github.com/gncs/botnet and was used in conjunction with version 0.3.2 of the e3nn library^63^ and version 1.8.0 of PyTorch^64^. If the code is not explicitly specified (in the benchmark section of the Supplementary Information), we used the eponymous code for each model.
