# Supplementary material for: The design space of E(3)-equivariant atom-centred interatomic potentials
Source: Nat Mach Intell. 2025 Jan 15;7(1):56–67. doi: 10.1038/s42256-024-00956-x (PMC11769842; doi:10.1038/s42256-024-00956-x)
Supplement: Supplementary file 1 — Supplementary benchmark results, Supplementary Figs. 1–7, Tables 1–6 and text. [file 42256_2024_956_MOESM1_ESM.pdf]

nature machine intelligence

Supplementary information

https://doi.org/10.1038/s42256-024-00956-x

The design space of E(3)-equivariant atom-centred interatomic potentials

In the format provided by the authors and unedited

Supplementary Information

I. Benchmark Experiments

1. rMD17: Small molecules benchmark

The revMD17 dataset contains five different train test splits of 10 different small organic molecules [1]. Each of the splits comprises 1000 configurations for each molecule sampled randomly from a long *ab initio* molecular dynamics simulation carried out at 500 K. The task is to fit a force field model on the 1000 examples and report the mean absolute error (MAE) of the total energy and the force components. Table I on the left of the vertical solid line shows the MAE of different models from the literature trained on precisely this dataset. We show the models on the right of the solid vertical line for completeness, but it is important to note that they were trained on an earlier version of this dataset which proved to be noisy, with different train-test splits and DFT settings. Both BOTNet and NequIP models far outperform the other approaches in all the molecules.

2. 3BPA: Extrapolation to higher tempature

To test the extrapolation capabilities of the different models to out-of-distribution input data we used the 3BPA dataset. This is well suited as the molecule is flexible with 3 rotating bonds as illustrated on Figure 1. We use the training set collected at 300K because it samples only the pockets corresponding to the most stable dihedral angle combinations, whereas the higher temperature test sets contain geometries sampling the full dihedral profile [3].

The root-mean-squared errors (RMSE) on energies and force components of several different models are shown in Table II. We have included the RMSE-s on the three different temperature test sets and a 4th test set made up of the DFT dihedral scan geometries. The 300K test set measures the in-domain accuracy of the models. We observe similar accuracy between BOTNet and NequIP within the standard deviation, outperforming the closest model linear ACE by a factor of 2. At 600K, we observe higher RMSE over all the models as the data are further from the training set and the magnitudes of the forces are also larger. Compared to the other models, BOTNet and NequIP are again more accurate by about a factor of 2. The 1200K test set measures the most extreme extrapolation. In this case, the BOTNet model has the highest accuracy, performing around 20% better than NequIP and over two times better than all other models. BOTNet is again the most accurate model on the dihedral scan, a different set of out-of-domain samples, proving its excellent extrapolation capabilities compared to the other models.

We also inspect the shape of the potential energy surface by scanning along lines in the three-dimensional dihedral space, keeping α and β fixed and varying γ. The energy along three such cuts is plotted in Figure 1 showing BOTNet, NequIP, and linear ACE predictions. The three cuts present different degrees of prediction difficulty, primarily because they probe the PES at different energy levels above the equilibrium state. The $\beta = 120°$ on the left and the $\beta = 180°$ on the right of Figure 1 (b) are easier, because there are some training points in the dataset with similar combinations of dihedral angles, whereas the third cut in the middle ($\beta = 150°$) is the most challenging of the three, with no training data points near it.

We can observe that all three models tested perform similarly well on the two easier cuts, with NequIP being the most accurate. On the most challenging cut in the middle of Figure 1 (b), we see that linear ACE smoothly reproduces the shape of the potential energy but overestimates the rotation barriers by about a factor of two. Both NequIP and BOTNet can predict the overall shape and barrier height with remarkable accuracy, with BOTNet even getting the overall energy shift right. Overall, all three models perform well on these tests, linear ACE is extrapolating smoothly, but very far from the training set, it can make more significant errors. In contrast, the nonlinear models are smooth and accurate even for input data far from the training distribution.

3. Acetylacetone: flexibility and reactivity

The potential energy surface of acetylacetone has been studied exhaustively in the past due to its many interesting properties, such as the tunneling splitting of the intramolecular hydrogen transfer [12]. In this paper, we are not trying to create the most accurate PES of the molecule but deliberately use a small training set, making the task challenging for the inference methods. This helps us see the distinctions between the different models. To prepare the training set, we run a long molecular dynamics simulation at 300 K using a Langevin thermostat at the semi-empirical GFN2-xTB level of theory. We sampled several independent configurations and computed the energies and forces with density functional theory using the PBE exchange-correlation functional with D3 dispersion correction and def2-SVP basis set using the ORCA electronic structure package.

To test the models, we measure extrapolation both in temperature and along two internal coordinates of the molecule. The temperature extrapolation experiments show similar results to the 3BPA case, though NequIP performs slightly better than BOTNet. The results are shown in Table III.

Figure 2 shows the predictions of the three models

along two different internal coordinates. The left panel shows the energy change as a function of one of the O-C-C-C dihedral angles. The training set only samples dihedral angles below 30°, and we test the models on angles up to 180°. This is a significant extrapolation in input space and energy space as the rotation barrier is about 1 eV, much more significant than the typical energy fluctuations in the training set. All models produce a smooth PES, reproducing the maxima at around 90 degrees, but remarkably NequIP and BOTNet also get the height of the barrier very accurately. We also observe that NequIP achieves better reproduction of the PES after the maxima.

On the right panel of Figure 2 we plot the energy along a reaction coordinate of the intramolecular hydrogen transfer found using the Nudged Elastic Band method [13]. This task probes how the models can cope with reactivity not too far from the training set. We can see that all models reproduce the shape of the barrier accurately, with BOTNet and NequIP getting the height of the barrier within 2 meV.

II. BOTNet: Body Ordered Equivariant Network

The design space of the Multi-ACE framework provides a setting to study the choices made by different approaches. The most accurate model published to date is NequIP, which uses an equivariant 2-body message passing scheme. We probe the NequIP architecture to understand which parts are crucial for its success and study how changing different parts of the architecture affects the properties of the fitted potential energy surface, including smoothness and out-of-domain extrapolation. The model introduced in this section, BOTNet, is a simplified, body-ordered version of NequIP. We keep the two-body interactions of NequIP within each layer, and the body-order is increased by one in each iteration of the message passing. This is made possible by removing all pointwise non-linearities in the update, except in the last layer. The different body-ordered contributions to the total energy are predicted as a sum of functions of the learnable $t+1$-body features $h_i^{(t)}$ at each iteration. Note that the BOTnet model is still a nonlinear function of its parameters due to the tensor product operation in the message block.

The final energy expression of BOTnet can be written as a body-ordered energy expansion,

$$E_i = E_i^{(0)} + \sum_{t}^{T-1} W^{(t)} \boldsymbol{h}_i^{(t)} + \mathcal{F}\left(W^{(T)} \boldsymbol{h}_i^{(T)}\right) \qquad (1)$$

where $W^{(t)}$ are learnable weights representing a linear combination of the features giving the body-ordered energy terms and $\mathcal{F}\left(W^{(T)} \boldsymbol{h}_i^{(T)}\right)$ is a generic nonlinear function accounting for the residual higher order terms in the truncated expansion. The terms $\boldsymbol{h}_i^{(t)}$ have exactly correlation order t (body-order $t+1$).

The general body-ordered expansion is

$$F(\{r_i\}_{i=1}^N) = f^{(0)} + \sum_{i=1}^{N} f^{(1)}(r_i) + \sum_{1\leq i<j\leq N} f^{(2)}(r_i, r_j) \cdots + \sum_{1\leq i_1<\ldots<i_N\leq N} f^{(N)}(r_{(i_1)}, r_{(i_2)}, ..., r_{(i_N)}). \qquad (2)$$

In Figure 4, we illustrate the hierarchical energy decomposition learned by a BOTNet model for the intramolecular hydrogen transfer reaction of acetylacetone. In Eq. (2), the number of terms summed over grows with the correlation order, but for an efficient expansion the total size of each contribution should decrease. In the case of BOTNet, we can observe that the terms are decreasing in absolute values (even after summing them for each correlation order over all centers). The last term is bigger than the correlation order three and four since it is accounting for all higher-order terms in the truncated expansion.

Below, we give a detailed description of the architecture of BOTNet. It retains the most crucial elements of NequIP while introducing some new architectural features. The details of the two architectures are compared in Figure 3.

a. Chemical embedding block The first block of both BOTNet and NequIP is the chemical embedding. The elements z_i are mapped to vectors of lengths equal to the number of elements via one-hot encoding. The one-hot vectors are multiplied with a learnable weight matrix of size $N_{\text{elements}} \times N_{\text{channels}}$ outputting a learnable invariant feature vector for each atom corresponding to $\boldsymbol{h}_i^{(0)}$.

b. Radial embedding block The radial features are composed of a Bessel basis multiplied by a smooth polynomial cutoff denoted by $R_n(r_{ji})$. The radial embedding block outputs an array of size N_n for each edge corresponding to the values of Bessel functions of different frequencies.

c. Interaction blocks The interaction block takes as input the node features $\boldsymbol{h}_i^{(t)}$, the radial features R_n, the spherical harmonics $Y_{l_1}^{m_1}$, and the node attributes $\boldsymbol{\theta}_i$. First, the node features $\boldsymbol{h}_i^{(t)}$ undergo a learnable linear transformation to mix the k channels. Then, the radial features R_n are transformed together with the one-hot encoding $\boldsymbol{\theta}_i$ by a learnable bi-linear operation into the learnable radial basis, $R_{kl_1l_2L}^{(t)}(r_{ji})$. The interaction block of the first layer differs from the rest of the layers in a simple way: In the first layer, we use a standard MLP having R_n as input and outputting $R_{kl_1l_2L}^{(0)}(r_{ji})$. In all subsequent layers we use a bilinear map combining the radial features R_n and the chemical attribute $\boldsymbol{\theta}_j$ outputting the radial basis $R_{kl_1l_2L}^{(t)}(r_{ji})$. The reason for having two different radial features (for the first and all subsequent layers) is that at the first layer, the attributes

$\boldsymbol{\theta}_j$ are already present in the feature $\boldsymbol{h}_j^{(0)}$, which makes the element-dependent radial basis redundant.

A symmetrized tensor product operation forms the edges features (one-particle basis) between the learnable radial basis, the spherical harmonics, and the node features. The symmetrized tensor product operation first makes the tensor product between the different elements and then decomposes it into irreducible representations using Clebsch-Gordan coefficients. The edges features are summed over the neighborhood of each atom to produce a message on each atom.

For the update phase of the message passing, we use a linear transformation followed by a simplified self-connection for the first layer and a residual self-connection for all the subsequent ones.

d. Readout blocks After each update, a linear readout maps the invariant part of the learnable feature to the local state energy $E_i^{(t)} = \mathcal{R}^{(t)}(\boldsymbol{h}_i^{(t)})$. The last readout at iteration T, uses a nonlinear MLP to account for the higher orders terms in the truncated order expansion E_{res}.

In summary, there are no non-linearities present in the network to preserve body ordering, except at the last readout. We show below that the inclusion of the higher-order term in the expansion results is sufficient to recover the accuracy of a fully nonlinear model. We introduce a self-connection to preserve body order at the first round of message passing, ensuring that the network does not learn any non-body-ordered energy shift. We will also show that careful normalization induces a dramatic impact on extrapolation and is crucial for its in-domain accuracy as well.

1. *Self-Connection*

An essential and often neglected part of MPNN models is the self-connection. It is a mechanism used to mix information from the previous layer with the output of the current layer in a learnable way. The self-connection mechanism is fundamentally related to the residual architecture of convolutional neural networks [14].

In NequIP the general message passing operation of is chosen to be independent of the receiver (central) atom chemical element θ_i. The effect of this is that the successive message passing iterations "dilute" the chemical information of the central atom. NequIP has introduced a self-connection that re-injects chemical information about the central atom after each message passing step to overcome this issue. It is part of the update and has the form reminiscent of residual neural networks:

$$h_{i,kLM}^{(t+1)} = h_{ikLM}^{(t+1)} + \sum_{a\tilde{k}} W_{k\tilde{k}La} \theta_{i,a} h_{i,\tilde{k}LM}^{(t)}, \tag{3}$$

where $W_{k\tilde{k}La}$ is a learnable weight matrix of size $[N_{\text{channels}} \times N_{\text{channels}} \times L_{\max} \times N_{\text{elements}}]$ of the attribute θ_i which is in the case of NequIP the one hot encoding of the chemical type z_i of the central (receiver) atom.

When the residual update of Eq. (3) is applied after the very first message passing iteration, during the training, the initial feature $h_i^{(0)}$ which is independent of the atomic environment, gets updated. This results in the model learning a shift to the potential energy that is only dependent on the central atom but not on the environment. To ensure that the model has the correct limit for isolated atoms, this self-connection cannot be applied at the first update. An alternative simplified self-connection, implemented as a bi-linear map, serves the purpose of reinjecting chemical information but does not have the residual connection:

$$h_{i,kLM}^{(t+1)} = \sum_{a\tilde{k}} W_{k\tilde{k}La} \theta_{i,a} h_{i,\tilde{k}LM}^{(t)} \tag{4}$$

This simplified self-connection has the advantage that the features at the $t = 0$ layer do not enter the energy expression removing the learnable shift. This is advantageous if it is necessary to enforce that the model predicts the correct energy for isolated atoms. Table IV shows a comparison of NequIP and BOTNet models with the residual and simplified self-connection. It appears that the self-connection plays a crucial role in message-passing accuracy. Moreover, using the residual self-connection, the models can perform significantly better than no residual architecture. The `mix sc` BOTNet model has the simplified self-connection in the first update followed by the residual one. This architecture does not have an internal learnable shift and can match the performance of the entirely residual architecture closely. The issue of self connections does not arise in the case of linear ACE because the chemical elements explicitly index the basis functions.

2. *Numerical stability*

Numerical stability is of significant importance for computations involving interatomic potentials. It affects the smoothness of the PES and, consequently, the stability of geometry optimisation and accuracy of molecular dynamics simulations. In Figure 5 we show a potential energy slice as one of the bond-angles is varied in the 3BPA molecule. The figure shows NequIP models trained using PyTorch with 32 and 64-bit floats. Using the lower precision results in a piecewise linear unsmooth potential energy surface. By using higher precision, the smoothness of the potential energy surface is significantly improved. The same phenomenon was observed with BOTNet.

III. Normalization

This section inspects the influence of normalization both inside the network and on the data. We show that normalization plays a significant role in converging these

over-parametrized models that rely on stochastic gradient estimation. However, non-physical normalization can hurt the extrapolation of the models far away from the training set.

A. Internal Normalization

Internal normalization refers to all procedures applied to internal features and weights to make them respect some statistical properties. It is of crucial importance in the convergence of stochastic gradient-based optimization, and one of the most used examples is batch-normalization [15]. The first type of internal normalization is that of learnable features. In NequIP and BOTNet, spherical harmonics are normalized such that the second moment of features inside the network is close to 1:

$$||\mathbf{Y}_l(x)||^2 = 2l+1, \quad x \in S^2. \tag{5}$$

The learnable features at each stage should also follow the same statistical property at initialization

$$\left\langle h_{i,kLM}^{(t)} \right\rangle^2 \approx 1. \tag{6}$$

The underlying motivation for this normalization [16] comes from the assumption that the weights obey

$$\langle w_j \rangle = 0 \tag{7}$$
$$\langle w_j w_k \rangle = \sigma^2 \delta_{ij}, \tag{8}$$

such that the two first moments of the product $h_i^{(t)} \cdot w$ are functions of $\left\langle h_{i,kLM}^{(t)} \right\rangle^2$ only,

$$\left\langle h_i^{(t)} \cdot w \right\rangle = \sum_{kLM} \left\langle h_{i,kLM}^{(t)} \right\rangle \langle w_{kLM} \rangle = 0 \tag{9}$$
$$\left\langle \left(h_i^{(t)} \cdot w \right)^2 \right\rangle = \sigma^2 \sum_{kLM} \left\langle h_{i,kLM}^{(t)} \right\rangle^2. \tag{10}$$

Another crucial normalization is the message-normalization. As the message uses a sum operation, it gathers an average of neighbors' features. NequIP [17] proposed to normalise the sum by the square root of the average number of neighbors, so that $\lambda = \sqrt{\langle \#\mathcal{N}(k) \rangle_k}$ in the message function. We found that, for BOTNet, dividing the sum by the average number of neighbors $\langle \#\mathcal{N}(k) \rangle_k$ across the training dataset yields the best results.

In Table V, we compare NequIP and BOTNet models with and without the message normalization. We observe a significant effect of the message normalization on the performance, especially at high temperatures, being responsible for a decrease in the error of over 30%. As models with and without this normalization have the same expressiveness, they only differ in their learning dynamics during optimization. These results highlight how crucial internal normalization is for the convergence of stochastic gradient optimization.

B. Data Normalization

Data normalization is widely used in many areas of deep learning to accelerate the convergence of the optimization [18]. We define data normalization as a general transformation of the data prior to training. In the context of machine learning interatomic potentials, normalization can also play a unique role by constraining the data to obey correct physical limits, for example in the case of dissociation to atoms.

Given a data set of energies $\mathcal{D}_\mathcal{E} = \left\{E^i\right\}_{i=1}^N$ and forces $\mathcal{D}_\mathcal{F} = \{F^{i,j}\}_{i=1}^{N,K}$, where j is a multi-index running over $K = N_{\text{atoms}} \times N_{\text{coordinates}}$, the normalization operation is a function $\Phi : E \mapsto \hat{E}$ and by the conservation principle $\Phi' : F \mapsto \hat{F}$, which ensures that the transformed data $\hat{\mathcal{D}}_\mathcal{E}$ and $\hat{\mathcal{D}}_\mathcal{F}$ has some statistical properties (statistical normalization), or in the case of interatomic potentials one might want these transformations to obey certain physical properties (physical normalization) such as correct limit for isolated atoms.

The most widely used normalization schemes is standardization that we refer to as scale shifting (SSH), transforming the data as,

$$\hat{E} = \frac{1}{\alpha}\left(E - \mathbb{E}_{\mathcal{D}_\mathcal{E}}(E)\right) \tag{11}$$
$$\hat{F} = \frac{1}{\alpha} F, \tag{12}$$

with $\mathbb{E}_{\mathcal{D}_\mathcal{E}}(E)$ is the average of the energies across the training set and α can be chosen to be either the root mean square of the forces across the dataset (referred as SSH forces rms) or the standard deviation of the energies across the dataset. This ensures that the target energies have zero-mean and unit variance meaning that $\mathbb{E}_{\hat{\mathcal{D}}_\mathcal{E}}(E) = 0$. This normalization scheme has the property that the models have a non-physical offset of the potential energy surface. This means that the arbitrary shift of the potential energy does not correspond to the energy of the isolated atoms. This does not affect the simulations as long as no dissociation to atoms is involved, for example, in bulk simulations, but can be problematic for reactive force fields.

The physical normalization can be written as:

$$\hat{E} = \frac{1}{\alpha}\left(E - \sum_{i=1}^{N} E_{0,Z_i}\right) \tag{13}$$
$$\hat{F} = \frac{1}{\alpha} F, \tag{14}$$

where N is the number of atoms in the molecule, α is a scaling factor (can be interpreted as a change of units), and E_{0,Z_i} is the atomic energy of the chemical element Z_i. Atomic energies are usually computed using the isolated energy method of the reference or using a linear regression on a dataset. This approach ensures that the dissociated limit with no-interaction energy is correct.

In the following, we are testing the effect of data normalization on both accuracy and extrapolation capabilities. We compare the performance of models with different data normalization in table VI on the 3BPA data set. We observe that models learning from scale-shifted data achieve the best accuracy, including at higher temperatures. This difference stems from the radically different learning tasks between the scale-shifted and the physically normalized models. The SSH models are learning to reproduce a narrow part of the potential energy surface near equilibrium. In contrast, the physically normalized models are constrained to obey limits far from the data distribution.

The right panel of Figure 6 shows the energy predicted by each of the models and the ground truth DFT as the O-H bond distance is varied with the position of the other atoms being fixed. The configuration space sampled in the training set along the O-H bond is very narrow, making this a challenging extrapolation task. As the H is moved far from the oxygen, the model has to predict the energy of the ethyl radical, which is not in the training set, and therefore predicting the exact energy is almost impossible. Nonetheless, it is still valuable to compare how smooth and physical the shape of the PES is. Overall, the models reach good fidelity to the DFT results near the equilibrium where most of the training data is. As expected, the scale-shifted models (NequIP SSH and BOTNet SSH) give nonphysical results far from the training set. The barrier height is about 1 eV which is largely underestimated compared to the DFT, which has a barrier of 4eV. The models with correct atomic energy at the limit (NequIP E0, BOTNet E0, and linACE) give a much better barrier and can predict remarkably accurately the energy of the radical. NequIP E0 and BOTNet E0 reproduce the potential energy surface between 1 Å and 2 Å with very high fidelity while there is no data in this area.

The two right panels of Figure 6 show the potential energy surface of ethanol as the atoms are moved along a low frequency ($874cm^{-1}$) and a high frequency ($3005cm^{-1}$). The low-frequency mode probes a C-C bending mode with no bond breaking. We see that both scale-shifted and E0 models do equally well at the task, likely due to the absence of bond breaking. The high-frequency mode probes a C-H stretching mode, eventually getting close to bond breaking. We observe that scale-shifted models with the wrong limit predict the C-H stretching less accurately. In contrast, BOTNet and NequIP models with the correct limit can accurately predict the potential energy surface up to bond breaking, confirming that inputting the correct limit is crucial for reactive interatomic potentials.

IV. Generalized Clebsch-Gordan Coefficients

The generalised Clebsch-Gordan coefficients are defined as product of Clebsch-Gordan coefficients:

$$C^{LM}_{l_1m_1,..,l_nm_n} = C^{L_2M_2}_{l_1m_1,l_2m_2} C^{L_3M_3}_{L_2M_2,l_3m_3} ... C^{L_NM_N}_{L_{N-1}M_{N-1},l_Nm_N} \tag{15}$$

and

$$L \equiv (L_2, .., L_N), \quad |l_1 - l_2| \le L_2 \le l_1 + l_2 \quad \text{and} \\ |L_{i-1} - l_i| \le L_i \le L_{i-1} + l_i \quad \forall i \ge 3 \tag{16}$$

$$M \in \{m_i | -l_i \le m_i \le l_i\} \tag{17}$$

In equivariant networks it is usually more convenient to employ features in spherical coordinates. A *spherical tensor* $t^{l_1\cdots l_d}_{m_1\cdots m_\nu}$ transforms as

$$D^{l_1}_{k_1m_1} \cdots D^{l_d}_{k_dm_d} t^{l_1\cdots l_d}_{m_1\cdots m_d} \circ R = t^{l_1\cdots l_d}_{k_1\cdots k_d},$$

where $D^l = D^l(R)$ are the Wigner D-matrices, and a symmetrisation analogous may be performed to enforce this equivariance,

$$\mathbf{B}_{i,\mathbf{v},\mathbf{lm}} = \int_{O(3)} \left(\mathbf{D}^{\mathbf{l}}_{\mathbf{mk}} E^{\mathbf{l}}_{\mathbf{k}}\right) A_{i\mathbf{v}} \circ R \, dR, \tag{18}$$

where $\mathbf{D}^{\mathbf{l}}_{\mathbf{mk}} = D^{l_1}_{k_1m_1} \cdots D^{l_d}_{k_dm_d}$ and $E^{\mathbf{l}}_{\mathbf{k}}$ the canonical basis of d-dimensional tensors with indices $k_t = -k_t, \ldots, l_t$.

V. Equivariant non-linearities

The constraint of equivariance on non-linearities takes very different forms depending on the details of the group and can only be discussed case-by-case. A general practice is to observe that the equivariance constraint is always satisfied for invariant messages. Let $m^{(t)}_{00}$ be the channels of invariant messages. Then for $U(R) = I, \forall R \in SE(3)$:

$$m^{(t)}_{00}(R[r_{i_1}, ..., r_{i_n}]) = m^{(t)}_{00}(r_{i_1}, ..., r_{i_n}), \forall R \in SE(3) \tag{19}$$

So the application of any non-linearity $\mathcal{F}$ will be

$$\mathcal{F}(m^{(t)}_{00}(R[r_{i_1}, ..., r_{i_n}])) = \mathcal{F}(m^{(t)}_{00}(r_{i_1}, ..., r_{i_n})) \tag{20}$$

For general equivariant channels LM of a message, the trick is to use square-norm gated non-linearities [19] of the form

$$\mathcal{F}\big(m^{(t)}_{LM}(r_{i_1}, ..., r_{i_n})\big) = \\ \mathcal{F}\big(\|m^{(t)}_{LM}(r_{i_1}, ..., r_{i_n})\|^2\big) m^{(t)}_{LM}(r_{i_1}, ..., r_{i_n}) \tag{21}$$

As the non-linearity is only applied to the squared norm of a feature which is always an invariant scalar, this type of nonlinear functions preserve equivariance. While taking the norm of the message does increase the body order by one, it is not doing so in a complete way as many other paths from equivariant features to invariants are ignored.

VI. Body-ordering of the SiLU non-linearity

Assume that

$$\mathcal{F}(x) = \text{SiLU}(x) = \frac{x}{1+e^{-x}}. \tag{22}$$

The Taylor expansion of SiLU can can be written as,

$$\text{SiLU}(x) = \frac{x}{1+\sum\limits_{k}^{+\infty}(-1)^k \frac{x^k}{k!}} = \sum_{k=0}^{+\infty} \frac{(-1)^k(2^n-1)B_n}{n} x^n \tag{23}$$

where B_n corresponds to Bernoulli numbers. One can immediately notice that

$$\frac{\partial^T \text{SiLU}(x)}{\partial x^T} \neq 0 \quad \forall T. \tag{24}$$

Thereby formally $T = +\infty$, and the resulting h_i^{t+1} will admit infinite body order.

VII. Alchemical learning - dimer curves

We have tested this alchemical learning by plotting the 2-body dimer dissociation of different chemical element combinations, as inferred from the ethanol and methanol dataset. This dataset contains complete molecules of ethanol and methanol. Both have a single oxygen atom; thus, the training set does not contain any configurations with two or more oxygen atoms. The dimers curves of linear ACE, NequIP, and BOTNet are shown in Figure 7. Our linear ACE implementation has no chemical embedding, and thus the O-O dissociation curve is identically zero. In contrast, NequIP and BOTNet predict the shape of the curves and the position of the minimum in a chemically sensible way. Since no dimers were in the training set, we do not expect to recover these dissociation curves with accuracy. However, the general shape and particularly the repulsive interaction for small interatomic distances is essential for obtaining stable molecular dynamics.

VIII. Description of data files for benchmark experiments

A. Ethanol and Methanol

The ethanol and methanol dataset has two training sets. The first is taken from the revMD17 dataset [1] and was sampled from a long 500 K *ab initio* molecular dynamics trajectory. The models trained on this dataset can be evaluated on an independent test set coming from the same distribution, i.e., decorrelated parts of the same molecular dynamics trajectory. Further, they can be tested for bond breaking extrapolation, by removing the hydrogen of the alcohol group and keeping the rest of the molecule fixed. Finally, we can test extrapolation by computing the energy change as atoms are displaced along a high- and a low-frequency normal mode from the optimal geometry.

The second training set contains the 1000 ethanol geometries of the first training set but is augmented by 300 methanol geometries also sampled from 500 K *ab initio* molecular dynamics simulation. The models trained using this mixed dataset can be used to analyse the 2-body component of the potentials. Having two different molecules is required for this analysis because it eliminates the possibility for the models to distribute the total energy amongst the bonds arbitrarily by having two molecules where the ratio of the number of bonds between given element pairs is different.

B. 3BPA

The 3BPA dataset contains snapshots of a large flexible drug-like organic molecule sampled from different temperature molecular dynamics trajectories [3]. The models can be trained either on 300 K snapshots or on mixed T snapshots sampled from 300 K, 600 K, and 1200 K. There are three independent test sets for each temperature. The models can also be tested on the challenging task of computing the energy along dihedral rotations of the molecule. This test directly probes the smoothness and accuracy of the part of PES that determines which conformers are present in a simulation, and hence has a direct influence on properties of interest such as binding free energies to protein targets. In the following experiments, we train models on 500 configurations sampled at 300K only and test on the three temperatures.

C. Acetylacetone

The potential energy surface of acetylacetone has been studied exhaustively in the past due to its many interesting properties, such as the tunneling splitting of the intramolecular hydrogen transfer [20]. In this paper, we are not trying to create the most accurate PES of this molecule but deliberately use a small training set of 500 configurations making the task particularly challenging. This helps us see the distinctions between the different models. To prepare the training set, we ran a long molecular dynamics simulation at 300 K using a Langevin thermostat at the semi-empirical GFN2-xTB level of theory [21]. We sampled configurations at an interval of 1 ps and re-computed the resulting set of con-

figurations with density functional theory using the PBE exchange-correlation functional with D3 dispersion correction and def2-SVP basis set and `VeryTightSCF` convergence settings using the ORCA electronic structure package. To test the models, we measure extrapolation both in temperature and along two internal coordinates of the molecule, the hydrogen transfer path and a partially conjugated double bond rotation, which has a very high barrier for rotation.

[1] A. S. Christensen and O. Anatole von Lilienfeld, Machine Learning: Science and Technology **1** (2020), 10.1088/2632-2153/abba6f.

[2] S. Chmiela, A. Tkatchenko, H. E. Sauceda, I. Poltavsky, K. T. Schütt, and K.-R. Müller, Science advances **3**, e1603015 (2017).

[3] D. P. Kovács, C. v. d. Oord, J. Kucera, A. E. A. Allen, D. J. Cole, C. Ortner, and G. Csányi, Journal of Chemical Theory and Computation **17**, 7696 (2021), pMID: 34735161, https://doi.org/10.1021/acs.jctc.1c00647.

[4] F. A. Faber, A. S. Christensen, B. Huang, and O. A. von Lilienfeld, The Journal of Chemical Physics **148**, 241717 (2018), https://doi.org/10.1063/1.5020710.

[5] V. L. Deringer, A. P. Bartók, N. Bernstein, D. M. Wilkins, M. Ceriotti, and G. Csányi, Chemical Reviews **121**, 10073 (2021), pMID: 34398616, https://doi.org/10.1021/acs.chemrev.1c00022.

[6] X. Gao, F. Ramezanghorbani, O. Isayev, J. S. Smith, and A. E. Roitberg, Journal of Chemical Information and Modeling **60**, 3408 (2020), pMID: 32568524, https://doi.org/10.1021/acs.jcim.0c00451.

[7] K. T. Schütt, O. T. Unke, and M. Gastegger, CoRR **abs/2102.03150** (2021), 2102.03150.

[8] V. Zaverkin and J. Kästner, Journal of Chemical Theory and Computation **16**, 5410 (2020).

[9] J. Klicpera, J. Groß, and S. Günnemann, "Directional message passing for molecular graphs," (2020), arXiv:2003.03123 [cs.LG].

[10] M. Haghighatlari, J. Li, X. Guan, O. Zhang, A. Das, C. J. Stein, F. Heidar-Zadeh, M. Liu, M. Head-Gordon, L. Bertels, *et al.*, Digital Discovery **1**, 333 (2022).

[11] K. Schütt, P.-J. Kindermans, H. E. Sauceda Felix, S. Chmiela, A. Tkatchenko, and K.-R. Müller, in *Advances in Neural Information Processing Systems*, Vol. 30, edited by I. Guyon, U. V. Luxburg, S. Bengio, H. Wallach, R. Fergus, S. Vishwanathan, and R. Garnett (Curran Associates, Inc., 2017).

[12] C. Qu, P. L. Houston, R. Conte, A. Nandi, and J. M. Bowman, The Journal of Physical Chemistry Letters **12**, 4902 (2021), pMID: 34006096, https://doi.org/10.1021/acs.jpclett.1c01142.

[13] B. J. Berne, G. Ciccotti, and D. F. Coker, *Classical and Quantum Dynamics in Condensed Phase Simulations* (WORLD SCIENTIFIC, 1998) https://www.worldscientific.com/doi/pdf/10.1142/3816.

[14] K. He, X. Zhang, S. Ren, and J. Sun, "Deep residual learning for image recognition," (2015), arXiv:1512.03385 [cs.CV].

[15] S. Ioffe and C. Szegedy, "Batch normalization: Accelerating deep network training by reducing internal covariate shift," (2015), arXiv:1502.03167 [cs.LG].

[16] M. Geiger, T. Smidt, A. M., B. K. Miller, W. Boomsma, B. Dice, K. Lapchevskyi, M. Weiler, M. Tyszkiewicz, S. Batzner, M. Uhrin, J. Frellsen, N. Jung, S. Sanborn, J. Rackers, and M. Bailey, "Euclidean neural networks: e3nn," (2020).

[17] S. Batzner, A. Musaelian, L. Sun, M. Geiger, J. P. Mailoa, M. Kornbluth, N. Molinari, T. E. Smidt, and B. Kozinsky, Nature Communications **13**, 2453 (2022).

[18] Y. Lecun, L. Bottou, G. B. Orr, and K.-R. Müller, "Efficient backprop," (1998).

[19] M. Weiler, M. Geiger, M. Welling, W. Boomsma, and T. Cohen, CoRR **abs/1807.02547** (2018), 1807.02547.

[20] C. Qu, R. Conte, P. L. Houston, and J. M. Bowman, Phys. Chem. Chem. Phys. **23**, 7758 (2021).

[21] C. Bannwarth, S. Ehlert, and S. Grimme, Journal of Chemical Theory and Computation **15**, 1652 (2019), pMID: 30741547, https://doi.org/10.1021/acs.jctc.8b01176.

TABLE I. Mean absolute error on rMD17 dataset. Energy (E, meV) and force (F, meV/Å) errors of different models trained on 1,000 samples. The models on the left were trained and tested using the same train-test splits of rMD17 except for NequIP which uses 1,000 configuration sampled from the full rMD17 dataset [1], whereas models on the right use the original MD17 [2]. The best model for each molecule (on the left and the right) are shown in bold font. Bold numbers correspond to lowest error in each row. For reference 43 meV = 1 kcal / mol.

		BOTNet	NequIP	Linear ACE [3]	sGDML [3]	FCHL [4]	GAP [5]	ANI [6]	PaiNN [7]	GMsNN [8]	DimeNet [9]	NewtonNet [10]	SchNet [11]
**Aspirin**	E	**2.3**	**2.3**	6.1	7.2	6.2	17.7	16.6	6.9	16.5	8.8	7.3	16.0
	F	8.5	**8.2**	17.9	31.8	20.9	44.9	40.6	16.1	29.9	21.6	15.1	58.5
**Azobenzene**	E	**0.7**	**0.7**	3.6	4.3	2.8	8.5	15.9	-	-	-	6.1	3.5
	F	3.3	**2.9**	10.9	19.2	10.8	24.5	35.4	-	-	-	5.9	16.9
**Benzene**	E	**0.03**	0.04	0.04	0.06	0.35	0.75	3.3	-	3.5	3.4	-	-
	F	**0.3**	**0.3**	0.5	0.8	2.6	6.0	10.0	-	9.1	8.1	-	-
**Ethanol**	E	**0.4**	**0.4**	1.2	2.4	0.9	3.5	2.5	2.7	4.3	2.8	2.6	3.5
	F	3.2	**2.8**	7.3	16.0	6.2	18.1	13.4	10.0	14.3	10.0	9.1	16.9
**Malonaldehyde**	E	**0.8**	**0.8**	1.7	3.1	1.5	4.8	4.6	3.9	5.2	4.5	4.1	5.6
	F	5.8	**5.1**	11.1	18.8	10.3	26.4	24.5	13.8	19.5	16.6	14.0	28.6
**Naphthalene**	E	**0.2**	**0.2**	0.9	0.8	1.2	3.8	11.3	5.1	7.4	5.3	5.2	6.9
	F	1.8	**1.3**	5.1	5.4	6.5	16.5	29.2	3.6	15.6	9.3	3.6	25.2
**Paracetamol**	E	**1.3**	1.4	4.0	5.0	2.9	8.5	11.5	-	-	-	6.1	-
	F	**5.8**	5.9	12.7	23.3	12.3	28.9	30.4	-	-	-	11.4	-
**Salicylic acid**	E	0.8	**0.7**	1.8	2.1	1.8	5.6	9.2	4.9	8.2	5.8	4.9	8.7
	F	4.3	**4.0**	9.3	12.8	9.5	24.7	29.7	9.1	21.2	16.2	8.5	36.9
**Toluene**	E	**0.3**	**0.3**	1.1	1.0	1.7	4.0	7.7	4.2	6.5	4.4	4.1	5.2
	F	1.9	**1.6**	6.5	6.3	8.8	17.8	24.3	4.4	14.7	9.4	3.8	24.7
**Uracil**	E	**0.4**	**0.4**	1.1	1.4	0.6	3.0	5.1	4.5	5.2	5.0	4.6	4.5
	F	3.2	**3.1**	6.6	10.4	4.2	17.6	21.4	6.1	14.3	13.1	6.4	3.3

TABLE II. Root-mean-square error on 3BPA dataset. Energy (E, meV) and force (F, meV/Å) errors of models trained and tested on configurations of the flexible drug-like molecule 3-(benzyloxy)pyridin-2-amine (3BPA) collected at 300K. Standard deviations are computed over three runs (seeds) and shown (if available) in brackets. Bold numbers correspond to lowest error in each row.

		BOTNet	NequIP	Linear ACE	sGDML	GAP	FF	ANI	ANI-2x
300 K	E	3.1 (0.13)	**3.0** (0.2)	7.1	9.1	22.8	60.8	23.5	38.6
	F	**11.0** (0.14)	11.6 (0.2)	27.1	46.2	87.3	302.8	42.8	84.4
600 K	E	**11.5** (0.6)	11.9 (1.1)	24.0	484.8	61.4	136.8	37.8	54.5
	F	**26.7** (0.29)	29.4 (0.8)	64.3	439.2	151.9	407.9	71.7	102.8
1200 K	E	**39.1** (1.1)	49.8 (4.0)	85.3	774.5	166.8	325.5	76.8	88.8
	F	**81.1** (1.5)	97.1 (5.6)	187.0	711.1	305.5	670.9	129.6	139.6
Dihedral	E	**16.3** (1.5)	27.0 (4.2)	22.2	-	-	-	-	-
	F	**20.0** (1.2)	23.8 (2.4)	39.2	-	-	-	-	-

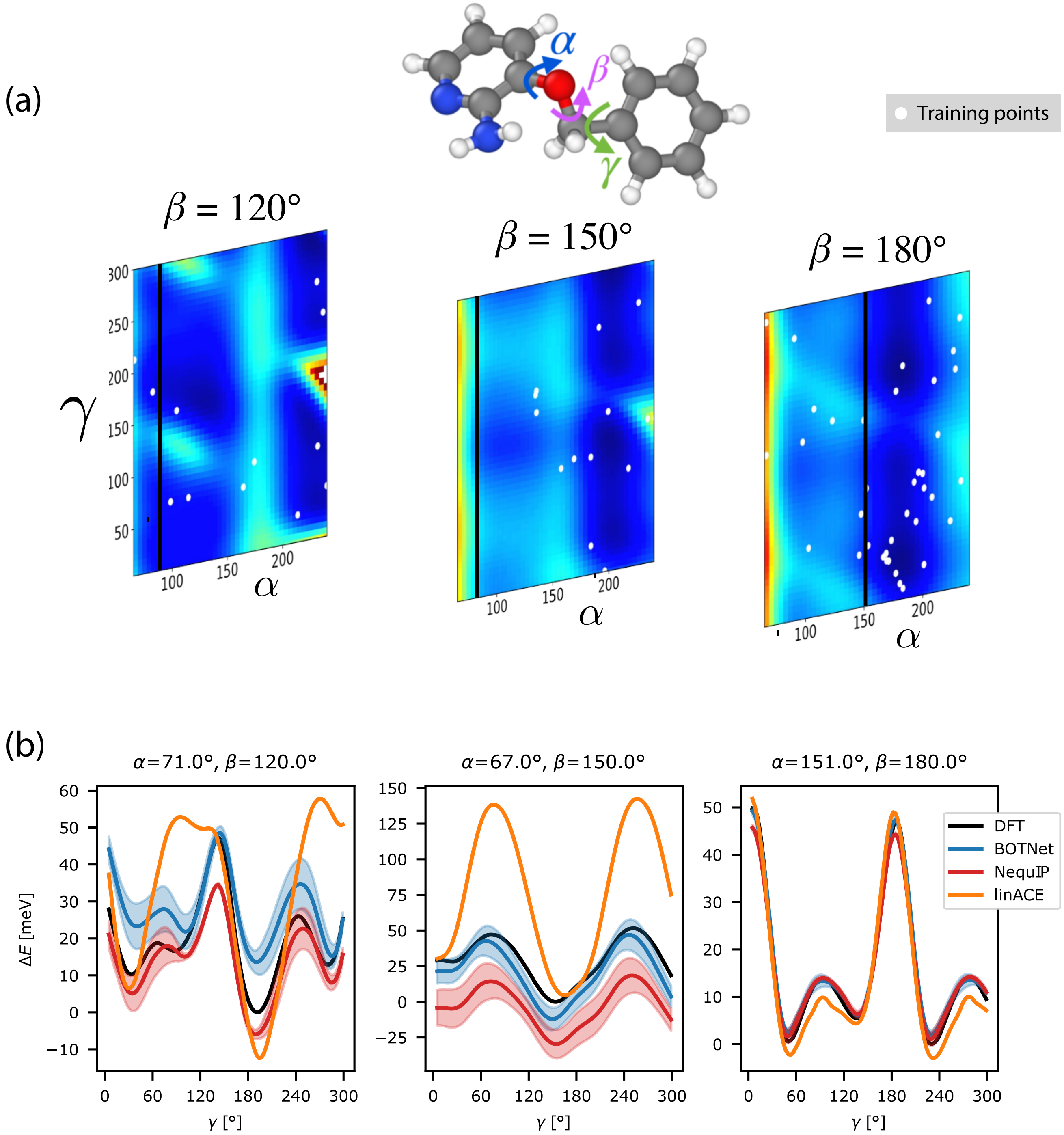

FIG. 1. **(a)** Two dimensional slices of the potential energy surface of 3-(benzyloxy)pyridin-2-amine (3BPA). Two of the three freely rotating dihedral angles (γ and α) vary between 0 and 300 degrees while the third β is kept fixed for each slice. The white dots corresponds to configuration in the training set at 300K that lie within $\pm 5^\circ$ of the fixed β. The black lines corresponds to the one dimensional cut of part (b) **(b)** Energy predictions on slices of the DFT potential energy landscape of 3-(benzyloxy)pyridin-2-amine (3BPA). The ground-truth (DFT) energy is shown in black. In all three panels, the energy scale is shifted so that the lowest point on the DFT curve is at zero. The shaded bands represent one standard deviation of an ensemble of models fitted with three different seeds, and the solid lines represent the mean.

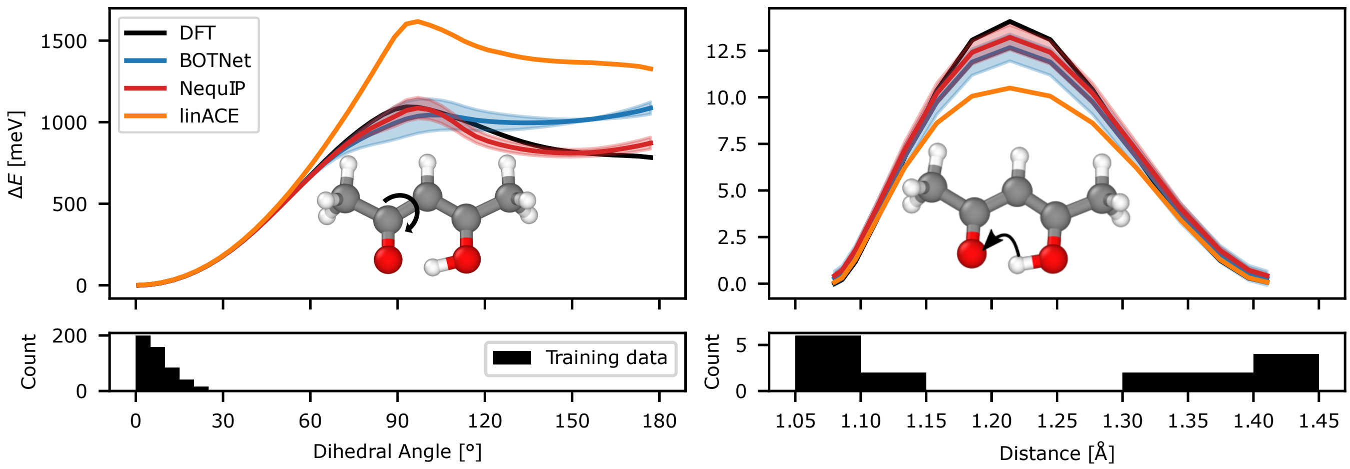

FIG. 2. On the left, mean energy predictions of a dihedral scan of the DFT potential energy landscape of Acetyl-Acetone. On the right mean energy prediction of the proton transfer in Acetyl-Acetone as a function of O-H distance. The shaded bands represent one standard deviation of an ensemble of models fitted with three different seeds, and the solid lines represent the mean.

TABLE III. Root-mean-square error on the acetylacetone dataset. Energy (E, meV) and force (F, meV/Å) errors of models trained on configurations of the acetylacetone molecule sampled at 300 K and tested on configurations sampled at 300 K and 600 K. Bold numbers correspond to lowest error in each row.

		BOTNet	NequIP	Linear ACE
300 K	E	0.89 (0.0)	**0.81** (0.05)	2.4
	F	6.3 (0.0)	**5.90** (0.46)	16.7
600 K	E	6.2 (1.1)	**6.04** (1.54)	8.3
	F	29.8 (1.0)	**27.8** (4.03)	41.8
Num Parameters		2,756,416	3,190,488	35,594

TABLE IV. Root-mean-square Energy (E, meV) and force (F, meV/Å) error on 3BPA dataset of NequIP without self connection (“no sc”) and with a fully residual self connection (“residual sc”), and BOTNet model with a fully residual self connection, only the simplified self connection (“simplified sc”) and with the use of a simplified self connection at the first layer and fully residual self connection in subsequent layers (“mixed sc”). As BOTNet and NequIP differ in many training settings, we bold both sides. Bold numbers correspond to lowest error in each row.

Model		NequIP residual sc	NequIP no sc	BOTNet residual sc	BOTNet mixed sc	BOTNet simplified sc
Code		nequip	nequip	botnet	botnet	botnet
Normalization		SSH forces rms	SSH forces rms	SSH forces rms	SSH forces rms	SSH forces rms
300 K	E	**3.0** (0.2)	3.8 (0.1)	**3.02**	3.1 (0.13)	3.7
	F	**11.6** (0.2)	15.8 (0.6)	11.7	**11.0** (0.14)	13.7
600 K	E	**11.9** (1.1)	17.1 (1.1)	12.3	**11.5** (0.6)	14.8
	F	**29.4** (0.8)	47.8 (2.9)	27.4	**26.7** (0.29)	37.1
1200 K	E	**49.8** (4.0)	108.5 (5.8)	43.5	**39.1** (1.1)	81.4
	F	**97.1** (5.6)	225.5 (14.0)	**79.9**	**81.1** (1.5)	126.93

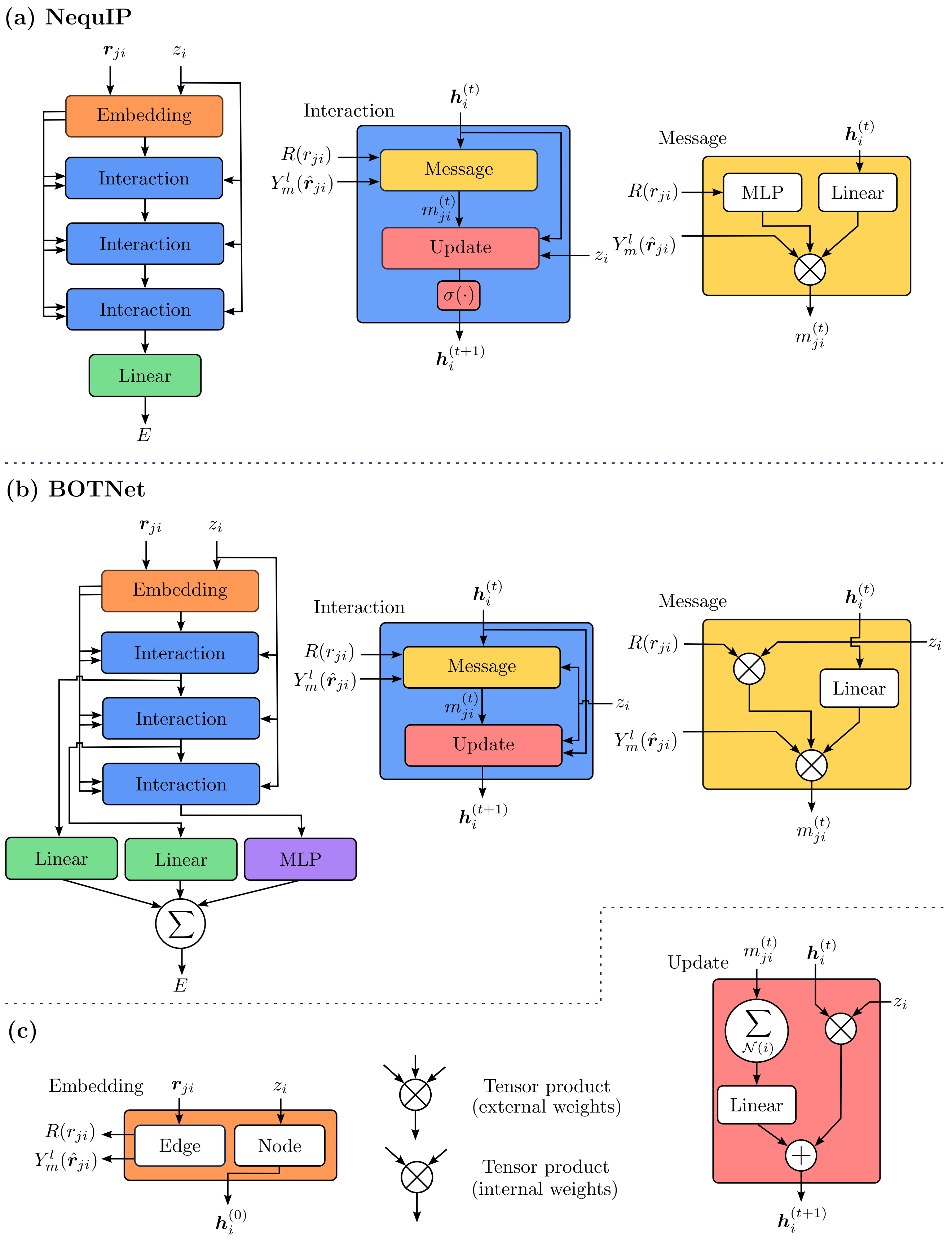

FIG. 3. Illustration of the architectures of NequIP (a) and BOTNet (b). Panel (c) contains illustrations of components the architectures have in common.

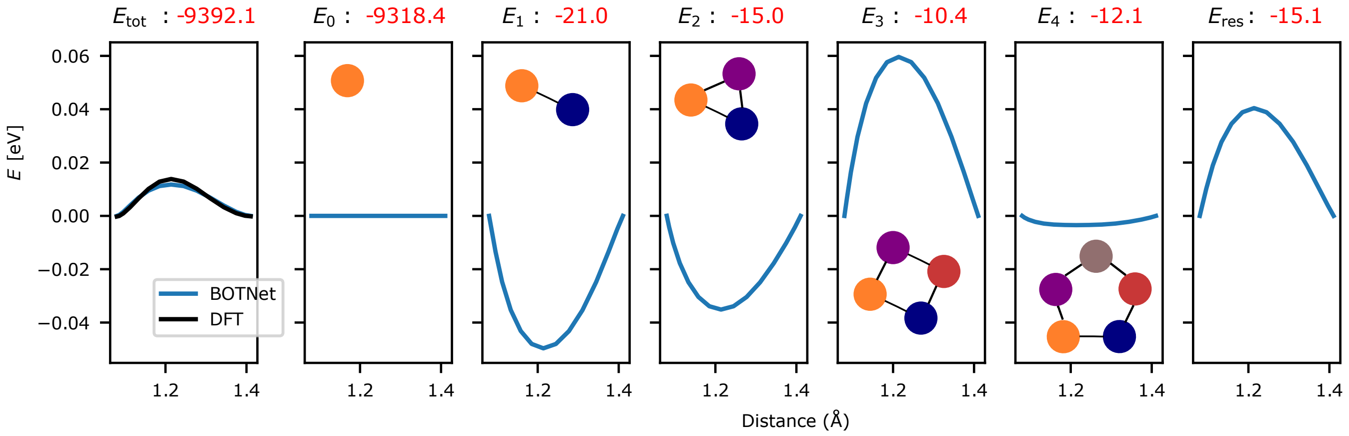

FIG. 4. Decomposition of the total energy predicted by BOTNet for the H-transfer pathway in Acetylacetone. E_0 corresponds to the "1-body" atomic energies. The contributions from E_1 to E_4 represent energies of increasing body order (2-body to 5-body, respectively). The curves are shifted by the energy of the last configuration in the transfer path, which is annotated in red above each plot. All energies are in eV.

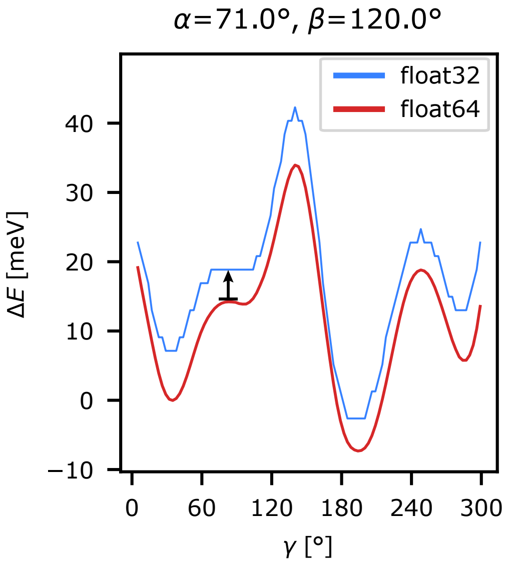

FIG. 5. **Floating point precision** Comparison of cuts of the 3BPA potential energy surface between NequIP models trained using 32 bit floats (red) and 64 bit floats (blue). The 32 bit curve was shifted upwards as indicated by the black arrow for better visibility.

TABLE V. Root-mean-square Energy (E, meV) and force (F, meV/Å) error on 3BPA dataset of NequIP and BOTNet models with different internal normalization. Bold numbers correspond to lowest error in each row.

		NequIP			BOTNet	
		no internal	$\sqrt{\langle \#\mathcal{N}(k) \rangle_k}$	$\langle \#\mathcal{N}(k) \rangle_k$	no internal	$\langle \#\mathcal{N}(k) \rangle_k$
Code		botnet	nequip	nequip	botnet	botnet
Normalization		SSH forces rms	SSH forces rms	SSH forces rms	SSH forces rms	SSH forces rms
300 K	E	3.3	3.0 (0.2)	**2.8**	3.5	3.1 (0.13)
	F	12.4	11.6 (0.2)	**10.8**	13.2	11.0 (0.14)
600 K	E	12.6	11.9 (1.1)	**10.6**	15.0	11.5 (0.6)
	F	33.3	29.4 (0.8)	26.8	38.8	**26.7** (0.29)
1200 K	E	54.6	49.8 (4.0)	43.1	89.6	**39.1** (1.1)
	F	117.6	97.1 (5.6)	85.5	138.5	**81.5** (1.5)

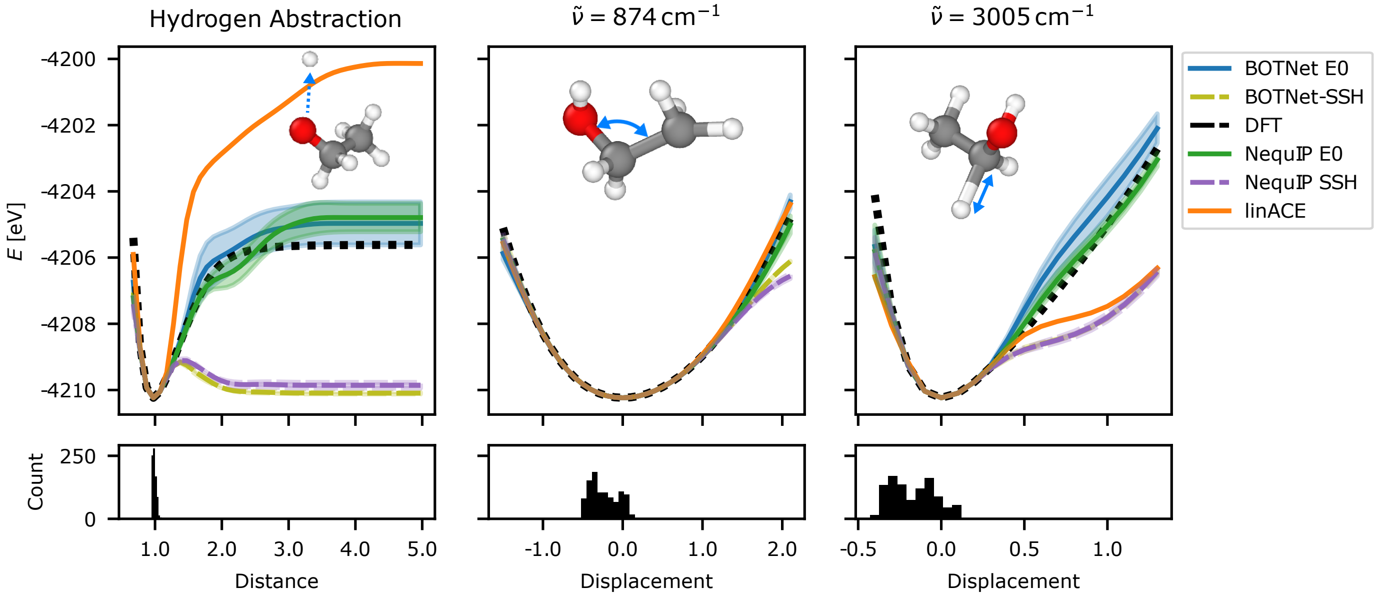

FIG. 6. Extrapolation capabilities of multiple models trained on the ethanol subset of the rMD17 dataset. Models that predict standardized energies (and forces) are shown with dashed lines. Note that linear ACE predicts non-standardized energies and forces. The ground-truth DFT energy is shown in black. **(a)**: predicted energy for a range of O–H bond lengths (cf. dashed blue arrow) while the remainder of the ethanol molecule is kept fixed. The bottom panel shows the distribution over the O–H bond lengths in the training dataset. **(b)** and **(c)**: predicted energy for molecular structures corresponding to displacements along two normal modes starting from the equilibrium structure (cf. blue arrows). The shaded bands represent one standard deviation of an ensemble of models fitted with three different seeds, and the solid lines represent the mean.

TABLE VI. Root-mean-square Energy (E, meV) and force (F, meV/Å) error on 3BPA dataset of NequIP and BOTNet models with different data normalization. Bold numbers correspond to lowest error in each row.

Model		NequIP	NequIP	BOTNet (Element Dependent)	BOTNet (Agnostic)	BOTNet
Code		nequip	nequip	botnet	botnet	botnet
Normalization		E_0	SSH forces rms	E_0	E_0	SSH forces rms
300 K	E	3.5	**3.0** (0.2)	3.6	3.5	**3.1** (0.13)
	F	13.0	**11.6** (0.2)	14.1	13.4	**11.0** (0.14)
600 K	E	13.4	**11.9** (1.1)	14.6	15.7	**11.5** (0.6)
	F	33.7	**29.4** (0.8)	35.7	33.5	**26.7** (0.29)
1200 K	E	55.1	**49.8** (4.0)	53.7	44.03	**39.1** (1.1)
	F	107.9	**97.1** (5.6)	111.4	101.7	**81.5** (1.5)

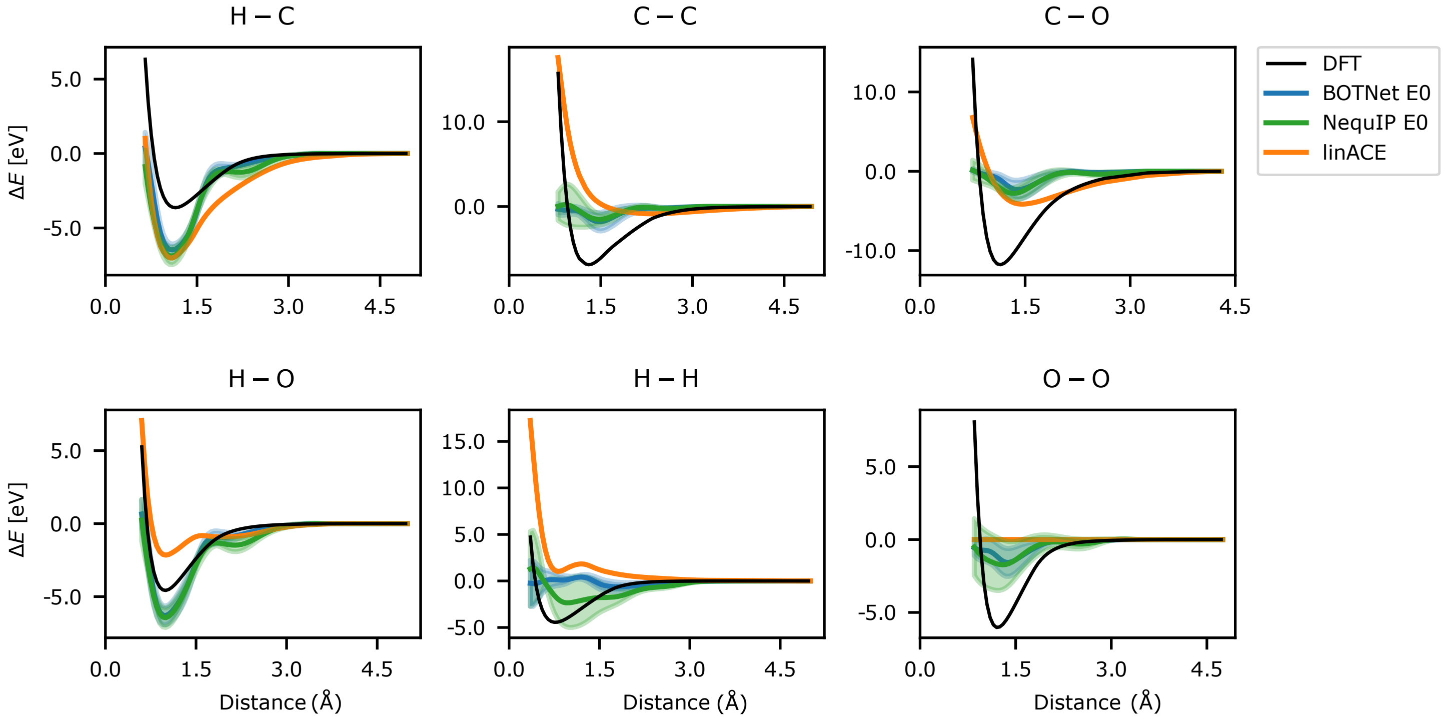

FIG. 7. Dissociation of dimers from chemical elements presents in ethanol and methanol. All dimers (combinations) apart from O–O dimers are present in the dataset. The shaded bands represent one standard deviation of an ensemble of models fitted with three different seeds, and the solid lines represent the mean.
